# Sox, Fox, and Lmx1b binding sites differentially regulate a Gdf5-Associated regulatory region during elbow development

**DOI:** 10.3389/fcell.2023.1215406

**Published:** 2023-07-10

**Authors:** Ruth-Love Yeboah, Charmaine U. Pira, Matthew Shankel, Allen M. Cooper, Endika Haro, Van-Dai Ly, Kenrick Wysong, Michael Zhang, Nicole Sandoval, Kerby C. Oberg

**Affiliations:** Department of Pathology and Human Anatomy, Loma Linda University, Loma Linda, CA, United States

**Keywords:** joint development, GDF5 gene, cis-regulatory module (CRM), LMX1b transcription factor, FOX transcription factor family, SOX transcription factors, osteoarthritis

## Abstract

**Introduction:** The articulating ends of limb bones have precise morphology and asymmetry that ensures proper joint function. Growth differentiation factor 5 (Gdf5) is a secreted morphogen involved in cartilage and bone development that contributes to the architecture of developing joints. Dysregulation of Gdf5 results in joint dysmorphogenesis often leading to progressive joint degeneration or osteoarthritis (OA). The transcription factors and *cis*-regulatory modules (CRMs) that regulate *Gdf5* expression are not well characterized. We previously identified a Gdf5-associated regulatory region (*GARR*) that contains predicted binding sites for Lmx1b, Osr2, Fox, and the Sox transcription factors. These transcription factors are recognized factors involved in joint morphogenesis and skeletal development.

**Methods:** We used *in situ* hybridization to *Gdf5*, *Col2A1*, and the transcription factors of interest in developing chicken limbs to determine potential overlap in expression. We further analyzed scRNA-seq data derived from limbs and knees in published mouse and chicken datasets, identifying cells with coexpression of *Gdf5* and the transcription factors of interest. We also performed site-directed mutatgenesis of the predicted transcription factor binding sites in a *GARR*-reporter construct and determined any change in activity using targeted regional electroporation (TREP) in micromass and embryonic chicken wing bioassays.

**Results:** *Gdf5* expression overlapped the expression of these transcription factors during joint development both by *in situ* hybridization (ISH) and scRNA-seq analyses. Within the *GARR* CRM, mutation of two binding sites common to Fox and Sox transcripstion factors reduced enhancer activity to background levels in micromass cultures and *in ovo* embryonic chicken wing bioassays, whereas mutation of two Sox-only binding sites caused a significant increase in activity. These results indicate that the Fox/Sox binding sites are required for activity, while the Sox-only sites are involved in repression of activity. Mutation of Lmx1b binding sites in *GARR* caused an overall reduction in enhancer activity *in vitro* and a dorsal reduction *in ovo*. Despite a recognized role for Osr2 in joint development, disruption of the predicted Osr2 site did not alter *GARR* activity.

**Conclusion:** Taken together, our data indicates that *GARR* integrates positive, repressive, and asymmetrical inputs to fine-tune the expression of *Gdf5* during elbow joint development.

## Introduction

The formation of joints is an important feature for skeletal movement and functionality. Limb joints are synovial in nature, having fluid-filled cavities that enable efficient motion. Synovial joint development begins as a compressed population of cells called an interzone that marks the future joint location within or at the distal end of condensing cartilage (Decker et al., 2014). This interzone region becomes a cavity between two abutting bones capped by articular cartilage and surrounded by a synovial membrane (Pitsillides and Ashhurst, 2008). Growth differentiation factor 5 (Gdf5) is an early marker of the interzone region (Archer et al., 2003; Jenner et al., 2014) and is recognized as an important factor for cartilage and synovial joint development (Francis-West et al., 1999; Storm and Kingsley, 1999; Buxton et al., 2001; Mikic, 2004; Mikic et al., 2004; Kania et al., 2020; Sun et al., 2021). Cells expressing *Gdf5* contribute to the various joint-associated tissues (i.e., articular cartilage, synovial membrane, ligament and tendon) (Shwartz et al., 2016; Bian et al., 2020).

Following specification of the various joint or joint-related structures, most cells downregulate *Gdf5* (Shwartz et al., 2016). An exception is articular chondrocytes, which maintain *Gdf5* expression into the postnatal period (Rountree et al., 2004; Decker et al., 2017). Movement refines the surfaces of the *Gdf5* positive articular cartilage (Kavanagh et al., 2006). It is noteworthy that a reduction in *Gdf5* expression during development is linked to a risk in subsequent articular cartilage degeneration [i.e., osteoarthritis (OA)] in later life (Miyamoto et al., 2007; Zhang et al., 2015; Hunter et al., 2020). Thus, regulation of *Gdf5* is critical for proper joint development (Miyamoto et al., 2007; Southam et al., 2007; Lettre et al., 2008; Sanna et al., 2008; He et al., 2015; Loughlin, 2015; Zhang et al., 2015; Chen et al., 2016; Zengini et al., 2018; Wojcik et al., 2019). We previously identified a *cis*-regulatory module (CRM) downstream of *Gdf5* via an Lmx1b-targeted ChIP-Seq (LBI443) that is active in limb joints during development; this confirmed another report which also identified this region as a CRM of *Gdf5* (Chen et al., 2016; Haro et al., 2017). However, the mechanisms through which this CRM control *Gdf5* expression and its differential maintenance in articular cartilage remain unclear.

There are several joint- and cartilage-associated transcription factors that could be potential regulators of *Gdf5* expression in synovial joint formation, including Sry box factors (Sox5, Sox6, Sox9, Sox4, Sox11) (Kan et al., 2013; Liu and Lefebvre, 2015), odd-skipped related factors (Osr 1 and 2) (Gao et al., 2011), and Lmx1b (Dreyer et al., 2004; Feenstra et al., 2012; Haro et al., 2017). Interestingly, these transcription factors have also been linked to OA and joint malformations (Lucas et al., 1966; Haag et al., 2008; Lee and Im, 2011; Curbo et al., 2019; Xu et al., 2019; Reynard and Barter, 2020). Forkhead box transcription factors (FoxC1 and FoxC2) regulate chondrocyte differentiation, and thus, may also regulate synovial joint development (Yoshida et al., 2015; Almubarak et al., 2021; Xu et al., 2021). FoxP transcription factors, which are involved in skeletal development and endochondral ossification, might also have a role in synovial joint development (Zhao et al., 2015; Xu P. et al., 2018). Herein, we demonstrate the overlapping expression patterns and single cell co-expression of these transcription factors with *Gdf5*. Furthermore, we provide data that suggests these transcription factors contribute to *Gdf5* regulation through the LBI443 CRM, renamed *Gdf5*-associated regulatory region (*GARR*), during synovial joint development in the elbow.

We use two model systems here: the developing chicken wing as an *in vivo* model and micromass cultures as an *in vitro* model system. Micromass cultures are 3D cultures of cartilage from mesenchymal cell (or limb mesodermal cells. It has been used extensively as a model for cartilage and bone growth (Mello and Tuan, 1999; Klumpers et al., 2015; Pirosa et al., 2019) and recently in joint-associated studies as well (Esmaeili et al., 2022; Ma et al., 2022; Salucci et al., 2022). In this study, we employed micromass cultures as a model for joint regulation through CRMs, and validated it as a suitable method to study *GARR* enhancer activity to enable more detailed mechanistic studies in the future.

## Methods

### *In silico* analysis

UCSC Genome Browser (RRID:SCR_005780) was used to determine *GARR* accessibility in the limb (limb ATAC-seq Open Chromatin track from ENCODE Regulation Pack) and conservation across placental mammals. The overview and conservation of the *Gdf5*-*GARR* locus were generated using Vista genome browser. CiiiDER (Gearing et al., 2019) was used to predict putative transcription factor binding sites in the *GARR* sequence using the JASPAR database of transcription factor binding motifs. For Lmx1b, the updated TMATWA binding motif was used to identify potential binding sites (Haro et al., 2017). The schematic diagram of binding sites was generated using the annotated sequence overview from Sequencher^®^ version 5.4.6 (Codes Corporation, Ann Arbor, MI).

### *In situ* hybridization

Whole mount and section *in situ* hybridization using digoxigenin-labeled mRNA probes to the transcription factors of interest was performed on Hamburger and Hamilton stage (HH) 27 chicken embryos as previously described (Yamada et al., 1999; Feenstra et al., 2012; Haro et al., 2021). Primers used for probe generation are listed in Supplementary Table S1. For section *in situ* hybridization, tissues were fixed in 4% PFA and paraffin embedded following standard procedure. Subsequently, 13 μm serial sections (8 μm for micromass cultures) were generated and processed as previously described (Moorman et al., 2001). Probe hybridization and washes were carried out at 60°C and 63°C, respectively. For all *in situ* targets, 3–5 embryos were examined.

### Immunofluorescence staining

Slides were deparaffinized and subjected to antigen retrieval for 20 min at 95°C using 10 mM citrate buffer (pH 6.0). Slides were washed and incubated in blocking buffer (10% fetal bovine serum and 1% BSA in TBST) for 1 h and then incubated at 4°C overnight humidified chamber with a mouse anti-GFP monoclonal antibody (Takara Bio, catalog no. 632381) at a 1:500 dilution in 1%BSA/TBST. Cells were subsequently washed with TBS and incubated with a fluorescently labeled donkey anti-mouse IgG Alexa Fluor™ 488 antibody (Invitrogen, catalog no. A21202) for 1 h at room temperature. After washing with TBS, nonspecific staining was removed with TrueBlack^®^ (Biotium, Fremont, California, United States) according to manufacturer’s protocol, and nuclei were stained using Hoechst dye. Slides were mounted with SlowFade™ gold antifade reagent (Invitrogen Waltham, MA) and imaged using confocal microscopy.

### Analysis of publicly available single cell data

Forelimb single cell RNA sequencing (scRNA-seq) data was obtained from He, P., Williams, B.A., Trout, D. et al., 2020 (He et al., 2020). The filtered h5 matrices were imported and processed using Partek^®^ Flow^®^ software, v10.0.23.0214 (RRID:SCR_011860). Cells with fewer than 600 transcripts and with more than 10% reads mapping to the mitochondrial genomes were filtered out. Samples were normalized as recommended using E_a,b_ = log2 [(CPM_a,b_) + 1], where CPM_a,b_ refers to counts-per-million for gene a, in sample b. Genes that were not detected in any cells were also filtered out. Differential expression analysis was performed on *Gdf5*-expressing (*Gdf5+*) cells (normalized expression greater than 0.5) *versus Gdf5* non-expressing (Gdf5-) cells (normalized expression lower than 0.5) using ANOVA. Analysis was also carried out with chondrogenic marker Col2A1 as an interaction term using both ANOVA and Hurdle [equivalent to MAST (Finak et al., 2015)] included in the Supplementary Tables S2–S8 and Supplementary Material. *p*-values were adjusted using FDR step-up as well as Bonferroni methods. Batch correction was used to minimize cross sample variation. PCA and tSNE analyses were conducted for dimensionality reduction and visualization of relationships among sequenced cells. Cell coexpressing Gdf5 and factors of interest were counted and expressed as a percentage (Supplementary Tables S9, S10).

### Site-directed mutagenesis of enhancer reporter constructs

Mouse *GARR*-GFP reporter constructs were generated from a thymidine kinase (tk) minimal promoter-driven GFP reporter (generous gift of Masanori Uchikawa). Transcription factor binding sites were disrupted using the QuikChange Lightning Site-Directed Mutagenesis Kit (Agilent Technologies, Santa Clara, CA) following manufacturer recommendations, and mutations were confirmed by Sanger sequencing. Briefly, core nucleotide sequences of the binding sites were modified to disrupt the binding site through primer design. We incorporated a restriction enzyme site in the mutated binding sites, when possible, for evaluation of successful mutagenesis. All potential binding site changes were evaluated by CiiiDER prior to mutation to ensure no new limb-relevant binding sites were inadvertently introduced. Primers used for site-directed mutagenesis are listed in Supplementary Table S11.

### Enhancer bioassay in chicken

Functional analyses of the *GARR*-GFP reporter constructs were performed by targeted regional electroporation into presumptive elbow mesoderm of HH23 chicken embryos (Oberg et al., 2002; Pira et al., 2008). Briefly, DNA cocktail containing 2 μg/μL GARR-GFP reporter construct, 0.2 μg/μL pCAGGS-RFP (to demonstrate transfection efficiency), 5% Fast green (to visualize the DNA cocktail), and TE buffer was injected (∼0.2 μL) into the limb bud mesoderm ∼600 μm from the distal tip using a glass microneedle and mineral oil for hydrolics. The DNA cocktail is chased with a small amount of mineral oil to seal the DNA into the mesodermal injection site. Sharpened tungsten electrodes (Omega Engineering, Stamford, CT) insulated with nail polish except for ∼200 μm of the tip was positioned to flank the DNA injection site. The anode was inserted into the mesoderm anterior to the DNA injection site, and the cathode was positioned posterior to the DNA site and only touching the surface of the limb bud. Electroporation was performed using the CUY21 Electroporator (Protech International, Boerne, TX) with 10 pulses of 35 V for a duration of 25 ms and with intervals of 50 ms. The embryos were harvested after 24 h, and GFP was visualized by digital image acquisition (Sony DKC-5000) into Adobe Photoshop (version 6.0, acquisition; version 2020, compilation).

### Micromass cultures

Forelimbs and hindlimbs of HH22–24 chicken embryos were collected, the ectoderm removed using trypsin (15 min s at 37°C), and cells pipetted to create a single cell suspension of mesodermal cells. Cells were seeded in 24-well culture plates in 10 μL volumes at a cell density of ∼400,000 cells. After about 2 h, cultures were flooded with 1 mL culture media containing DMEM supplemented with 10% FBS, 2% chicken serum, and Penicillin-Streptomycin (0.01 mg/mL). Cultures were followed as needed, changing the media every other day.

Cells to be used for micromass cultures were transfected as a single cell suspension (reverse transfection) using the Lipofectamine 3,000 reagent (Invitrogen, Waltham, MA). Transfection mixes were made using manufacturer recommendations for transfections in 24-well plates with some modifications. The total transfection mix volume was reduced to 5 μL by reducing the DMEM component. DMEM used for the transfection mixes had no FBS or antibiotic. Also, transfection was carried out without addition of the P3000 reagent. Cells were then added to the transfection mixes and seeded in 10 μL volumes (cell density of ∼400,000 cells maintained). After 2 h, culture media described above was added. Cultures were monitored for 36–48 h and then imaged using confocal microscopy or fluorescence microscopy.

### Image analysis and statistics

Fluorescence images were analyzed using FIJI (Schindelin et al., 2012). The RGB components (or channels) were split, and only the red and green channels were used for RFP and GFP fluorescence measurement, respectively. MaxEntropy threshold was used to outline region of interest (ROI) on greyscale (16bit) images. The mean intensity and area of ROIs were measured and used to calculate fluorescence intensity. GFP intensity was normalized to RFP intensity, and fold change in enhancer activity was analyzed by one-way ANOVA and Tukey’s HSD in GraphPad Prism (GraphPad ver. 9.0.0; San Diego, California) with α = 0.05. Results were displayed as swarm plots showing the range, interquartile range, and median. GraphPad *p*-value format was used; symbols used were **** (*p* < 0.0001), *** (*p* < 0.001), ** (*p* < 0.01), and * (*p* < 0.05).

## Results

### Multiple joint-related transcription factors are predicted to regulate *GDF5* expression through a *Gdf5*-associated regulatory region (*GARR*)

**FIGURE 1:**
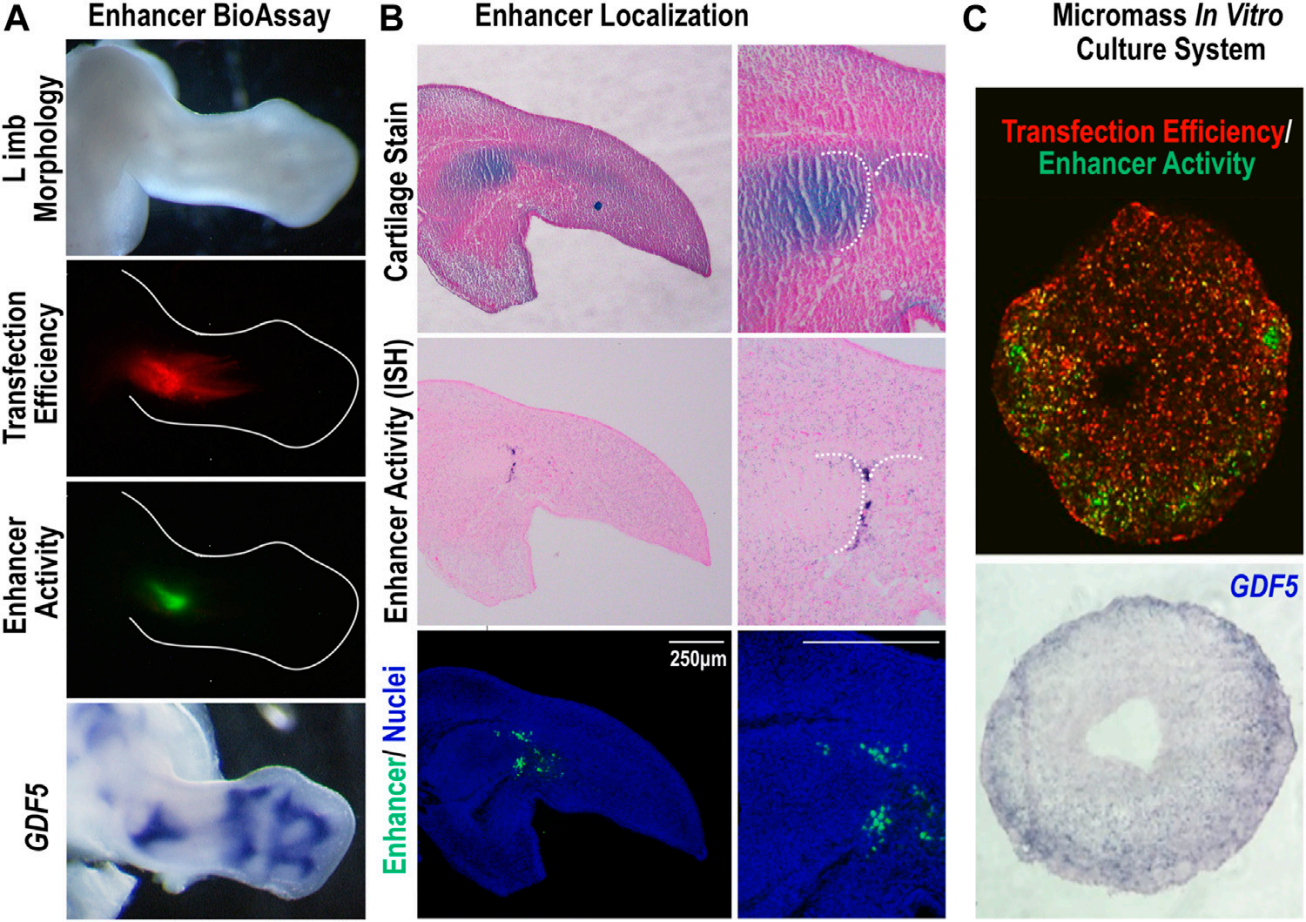
The *Gdf5*-Associated Regulatory Region (*GARR*) is active in joints of the limb **(A)** Targeted regional transfection of the *GARR*-reporter construct shows enhancer activity, indicated by GFP fluorescence, in the elbow joint of a chicken embryo, overlapping *GDF5* expression. Transfection efficiency is determined by a β-actin promoter driven RFP. **(B)** The cellular localization of *GARR* activity (GFP expression) by section *in situ* hybridization (middle panel). Immunofluorescent staining of the GFP reporter in an adjacent section; nuclei were stained with Hoechst dye (bottom panel). An adjacent section (top panel) was also stained with alcian blue and nuclear fast red to demonstrate the associated cartilage anlagen. **(C)** Micromass culture transfected with the *GARR-*reporter construct displaying activity (GFP fluorescence) accentuated around the periphery of the culture (upper panel). The activity correlates with *Gdf5* expression in a section *in situ* hybridization of the culture (bottom panel).

**FIGURE 2:**
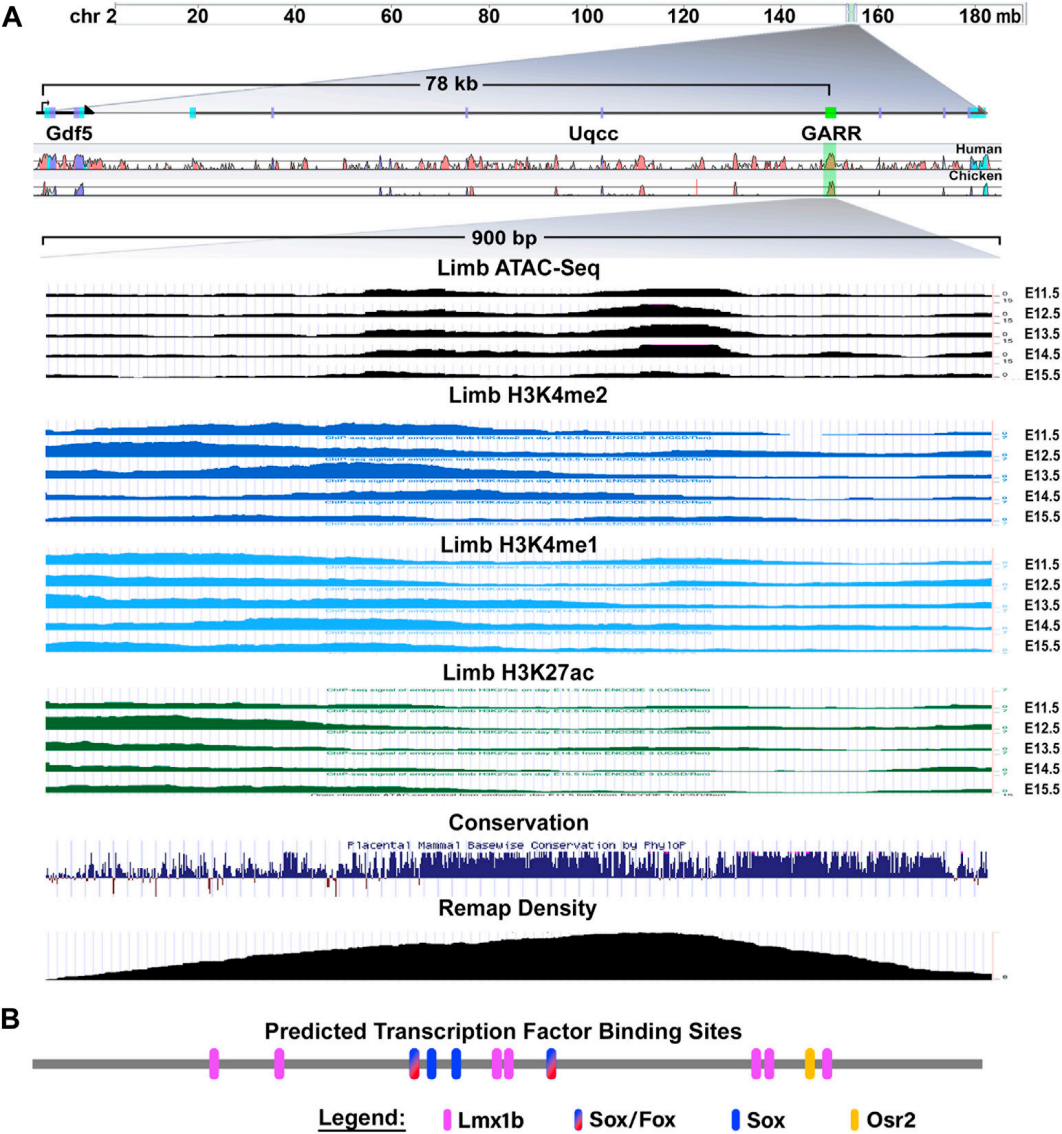
The Gdf5-Associated Regulatory Region *(GARR)* locus and characteristics **(A)** *GARR* is downstream of *Gdf5* in an intron of the *Uqcc1* gene. The region is accessible (determined by ATAC-seq), and has the chromatin features of a poised/active enhancer (determined by H3K4me1, H3K4me2 and H3K27ac) during joint development in the limb (E11–15). Multiple transcription factors bind to this region demonstrated by Remap ChIP track, and the region is highly conserved across vertebrates (image modified from UCSC genome browser view). **(B)** Schematic of joint- and cartilage-related transcription factors predicted (by CiiiDER) to bind to *GARR* (drawn to scale).

*GARR* is a *cis*-regulatory module (CRM) identified through an Lmx1b-targeted ChIP-seq analysis that shows activity in multiple joints within the limb (elbow, wrist, and digits) (Haro et al., 2017). Chen et al. (2016) demonstrated similar enhancer activity from a sequence corresponding to *GARR* (identified as R4), but with activity also in the shoulder*.* To confirm *GARR* activity, we performed targeted regional electroporation of a *GARR*-GFP reporter construct in the presumptive elbow of embryonic chicken wings. After 24 h, enhancer activity (GFP fluorescence) was observed in the elbow joint overlapping *GDF5* expression (Figure 1). This, together with the analysis of Hi-C data from mouse embryonic stem cells (Supplementary Figure S1), supports *GARR* regulation of *Gdf5.* Specifically, enhancer activity was observed both in joint spaces and in associated perichondrium (Figure 1B and Supplementary Figure S2) *In silico* evaluation of *GARR* shows that the 900 bp noncoding DNA sequence is located 82 Kb downstream of *Gdf5* in intron 7 (of 9) of the *Uqcc1* gene (Figure 2). *GARR* is highly conserved, and chromatin analysis (ATAC, acetylation, and methylation) shows *GARR* accessible and likely activation in the limb during early stages of joint formation/differentiation, i.e., embryonic days (E) 11–15 (Figure 2).

CiiiDER transcription factor binding site analysis using Jaspar 2020 database revealed conserved binding sites for transcription factors linked to cartilage and/or joint development, *Gdf5* regulation, and osteoarthritis (OA) pathogenesis (Figure 2B). There were four predicted Sox binding sites, two of which could also bind FoxC/P. A single Osr2 binding site was also predicted. There were also seven predicted Lmx1b sites: four were arranged as doublets (2bp gap between two sites) and three were single sites. Except for the single Lmx1b sites, all binding sites were conserved in placental vertebrates (see Supplementary Figures S3, S4). The high conservation and accessibility of the locus during joint formation imply they are reasonable candidates for mediating *GARR* activity.

### Colocalization of *GARR*-predicted transcription factors with *Gdf5* expression in limb cells

**FIGURE 3:**
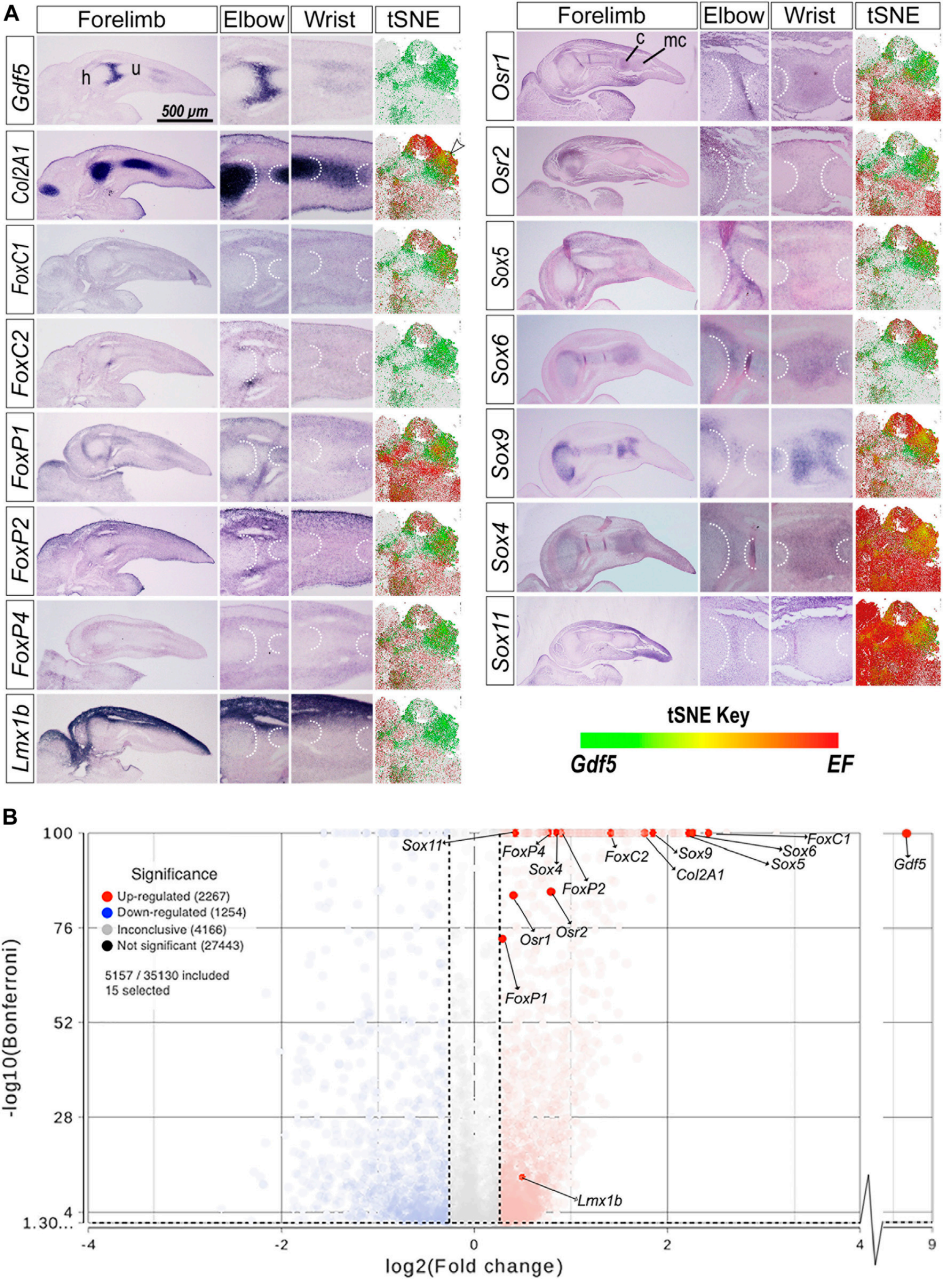
*Gdf5* expression overlaps with transcription factors with predicted binding sites on *GARR* **(A)** Section *in situ* hybridization (SISH) of *GDF5*, *COL2A1*, and transcription factors with predicted binding sites on *GARR*; aligned with t-distributed stochastic neighbor embedding (tSNE) plots, mapping scRNA-seq of mouse forelimb cells demonstrating colocalization (yellow/orange) of the *Gdf5 expressing* (*Gdf5*+) cell populations (green) and expressed factors (EF, red). **(B)** A volcano plot showing the differential expression of the predicted transcription factors in *Gdf5*+ cells compared to *Gdf5*-cells as analyzed by ANOVA. Left and right vertical dotted lines represent a+/-1.2-fold change of expression in Gdf5+ cells compared to *Gdf5-*cells. The horizontal dotted line near the bottom of the graph represents the Bonferroni adjusted *p*-value cutoff of 0.05. Abbreviations used: c (carpal), h (humerus), mc (metacarpal), and u (ulna). Arrowhead indicates colocalization of *Col2A1* and *Gdf5* in the tSNE plot.

To determine whether expression of transcription factors with predicted binding within *GARR* overlapped *Gdf5* expression, we performed whole mount and section *in situ* hybridization (WMISH and SISH, respectively) on chicken embryo forelimbs (Supplementary Figure S5 and Figure 3). Additionally, we examined the colocalization of *Gdf5* mRNA and the mRNA of predicted factors in published single-cell RNA sequencing data from mouse limbs (E11–15) (He et al., 2020) using t-distributed stochastic neighbor embedding (tSNE) plots (Figure 3). *Gdf5*, as expected, was expressed in the presumptive joint space of the elbow and wrist between cartilage anlagen demonstrated by *Col2A1*. The populations with *Gdf5*-expressing (*Gdf5+*) cells from the tSNE plots are shown with *Gdf +* cells in green (the full plots are included as Supplementary Figures S6 and S7). Cells expressing the candidate factors are red. When both *Gdf5* and these factors are present in the same cell it is yellow. For example, ISH reveals *Gdf5* expression in cartilage anlagen at the borders of a joint space overlapping *Col2A1* expression. The tSNE plot for *Col2A1* shows a subpopulation of cells in which *Col2A1* and *Gdf5* transcripts colocalize (yellow cells shown by arrowhead).

The expression of *FoxC1/2* and *FoxP1/2/4* was mostly in the perichondrium overlapping with *Gdf5* near the elbow and wrist joints in chicken limb. The tSNE plots showed areas of colocalization between *Gdf5* and the *Fox* transcription factors. Few cells expressed *FoxC2* in scRNA-seq; thus, colocalization in tSNE plots was not as evident as in the *FoxC1* tSNE plot. Similarly, the *FoxP1/2/4* factors overlapped *Gdf5* expression in the wrist and elbow of chicken forelimbs, and there was some colocalization in the tSNE plots in regions overlapping *Gdf5* and *Col2A1*-expressing cells (black arrowheads).

Lmx1b dorsalizes the limb, and its expression is restricted to dorsal limb mesoderm. *Lmx1b* had no substantial overlap with *Gdf5* or *Col2A1* expression within the cartilaginous anlagen or perichondrium. However, *Lmx1b* does overlap the dorsal aspect of the *Gdf5* expression at joint forming regions. Its limited, dorsally-restricted overlap with *Gdf5* is also evident in the tSNE plot for *Lmx1b* in which very limited colocalization is present in *Gdf5+* cells at the center of the plot (small black arrowhead). *Osr1/2* expression also overlapped with *Gdf5* at developing joints and colocalized with *Gdf5* in tSNE plots in a similar region as *Lmx1b*.

*Sox5/6/9* (known as the chondrogenic trio) and the *Sox4/11* (SoxC class) overlap with *Gdf5* expression. Since *Sox* transcription factors are expressed throughout the forming cartilage, there is some overlap with *Gdf5* which is predominantly expressed in cartilage. The *Sox* transcription factors also show high degree of colocalization with *Gdf5* in the tSNE plots. *Sox4* had the greatest extent of colocalization with *Gdf5* expression in the tSNE plots with nearly all *Gdf5+* cells coexpressing *Sox4*. *Sox9* expression was more robust than *Sox4* (throughout the cartilage in WMISH images) but had a similar colocalization with *Gdf5+* cells. Although there was also high colocalization of *Sox11* and *Gdf5* expression in the tSNE plots, *Sox11* was ubiquitously expressed in developing limb cells.

To further analyze the regulation potential for these transcription factors and the possible direction of regulation, we ran an ANOVA differential expression analysis between cells expressing *Gdf5* (*Gdf5+*) and those that do not (*Gdf5-*). The results are depicted in a volcano plot (Figure 3B) and tabulated in Supplementary Table S2. Statistical analysis of the scRNA-seq data shows a significantly higher expression of all predicted transcription factors in *Gdf5*+ cells. *FoxP1* was the least upregulated with a 1.22-fold increase. The Sox5/6/9 trio were upregulated 3–5-fold upregulated, while FoxC1 was over 5-fold higher in *Gdf5*+ cells. Similar patterns of upregulation are present in other published datasets (Supplementary Material) (Bian et al., 2020; Desanlis et al., 2020; Kelly et al., 2020).

In summary, the chosen Sox, Fox, Lmx1b and Osr transcription factors are expressed in and surrounding developing joints (images show the elbow and wrist). Their expression also colocalizes with *Gdf5*. This shows the potential for gene regulation as determined by the location of expression. Increased expression of *GARR*-predicted transcription factors in *Gdf5+* cells relative to *Gdf5-*cells implies the potential for a positive regulatory relationship with Gdf5 (Figure 3B). These data suggest that the *GARR*-predicted transcription factors promote *Gdf5* expression. Alternatively, transcription factors that overlap *GARR* activity could be regulated by Gdf5 signaling. However, since Gdf5 is a secreted factor, the response might not be accurately captured in *Gdf5+* cells.

### The absence of conserved Osr2 binding site does not alter *GARR* enhancer activity

**FIGURE 4:**
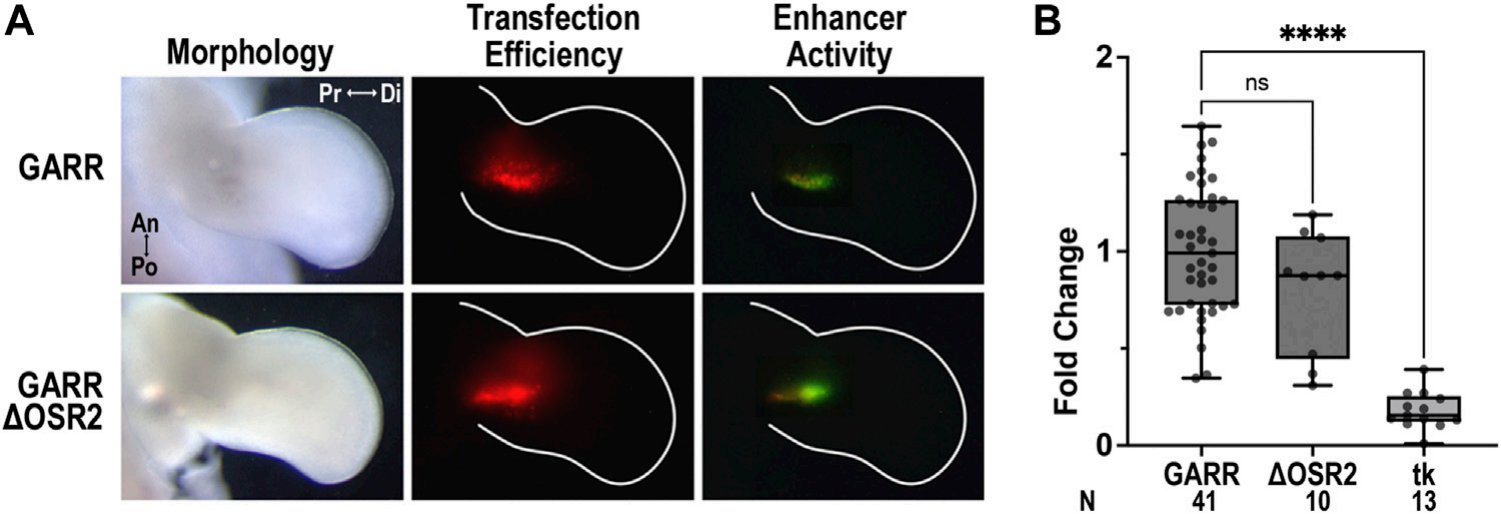
Conserved Osr2 binding site does not contribute to *GARR* activity in the developing chick elbow **(A)** Targeted transfection of the elbow with *GARR*-GFP containing the Osr2 binding site mutation (ΔOSR2) shows similar levels of activity compared to normal or wildtype *GARR* in a chicken bioassay. Transfection efficiency is indicated by RFP fluorescence. **(B)** Box plot of semi-quantification of fluorescent images from chick bioassay in A showing range, interquartile range, and median. Statistical significance is determined by one-way ANOVA and Tukey’s HSD (α = 0.05). Abbreviations used: an (anterior), di (distal), po (posterior), pr (proximal), and tk (tk-EGFP). N indicates the number of embryos.

We performed site-directed mutagenesis of the conserved binding sites in the mouse *GARR* sequence for transcription factors of interest to determine their impact on enhancer activity. Mutation of the single Osr2 binding site (ΔΟSR2) did not change *GARR* enhancer activity in the chicken elbow (Figure 4A). Semi-quantitative analysis of the fluorescence in images, reflective of enhancer activity, confirms this observation (Figure 4B). It is possible that other binding sites in *GARR* are used by Osr2, but our data suggest that this Osr2 binding site is insufficient to substantially alter *Gdf5’s* expression. Alternatively, Osr2 regulation of joint development may be through non-*Gdf5*-mediated pathways.

### Fox/Sox binding sites are necessary for *GARR* activity, whereas Sox-only sites convey repression

**FIGURE 5:**
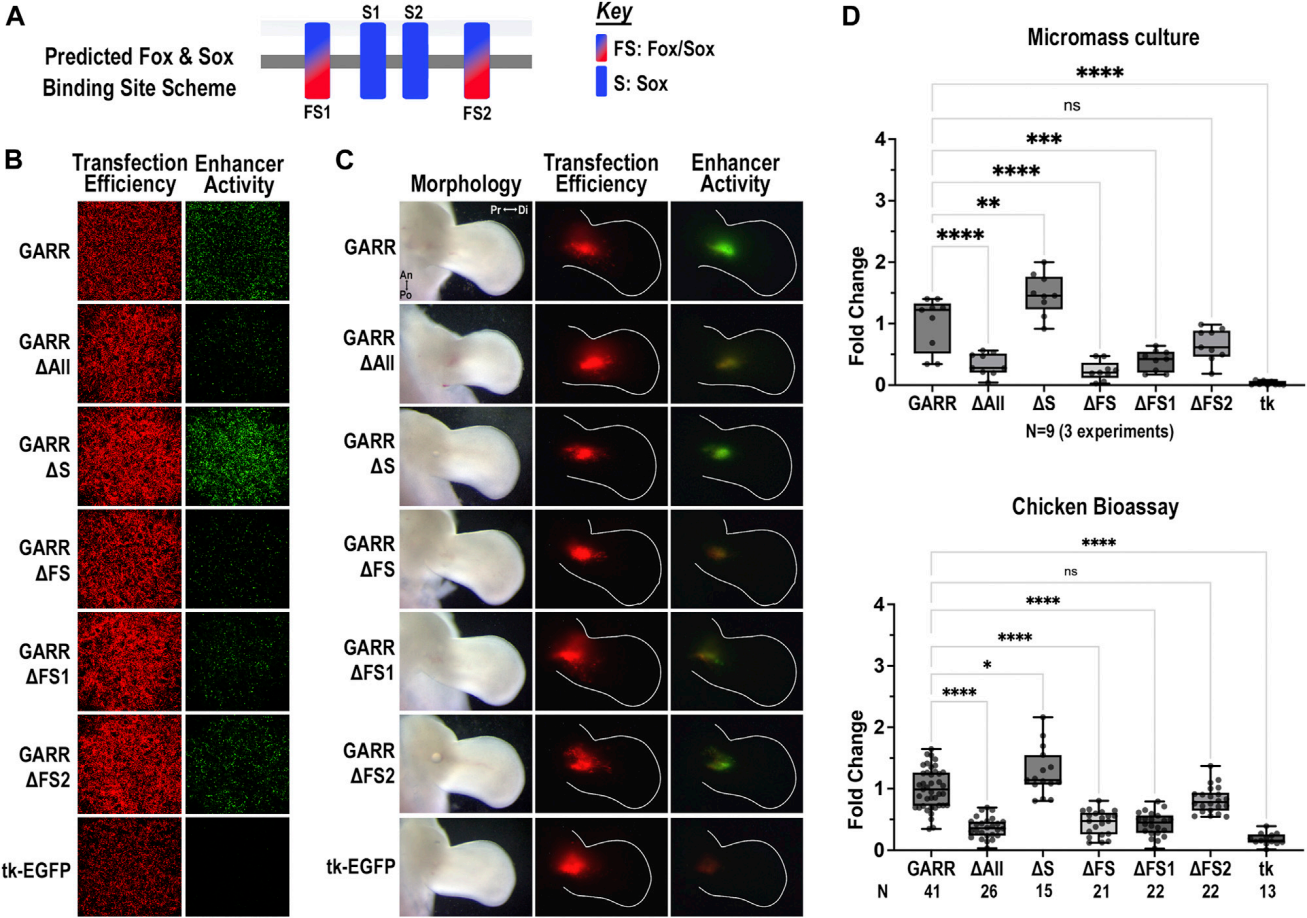
Differential regulation of *GARR* activity by Fox/Sox and Sox-only binding sites **(A)** Binding site scheme for shared Fox/Sox and Sox-only transcription factors (not to scale). **(B)** Micromass cultures transfected with various *GARR*-GFP reporter constructs with disrupted predicted binding sites for Fox/Sox (ΔFS1, ΔFS2, ΔFS for both sites), Sox-only (ΔS for both sites), and all Fox and Sox binding sites (ΔAll). ΔFS1, ΔFS and ΔAll show marked reduction, whereas ΔS shows an increase in enhancer activity **(C)** Targeted transfection of the elbow using the same *GARR*-GFP mutated constructs shows similar results. **(D)** Semi-quantification of fluorescent images from micromass and chick enhancer bioassay in B, C respectively. Box plots show range, interquartile range, and median with statistical significance determined by one-way ANOVA and Tukey’s HSD (α = 0.05). N indicates the number of experiments or embryos. Abbreviations used: an (anterior), di (distal), po (posterior), pr (proximal), and tk (tk-EGFP).

The mouse *GARR* sequence has two Fox/Sox sites and two Sox-only sites. In micromass cultures (Figure 4B), disruption of both Fox/Sox binding sites (ΔFS) reduced enhancer activity to background levels (compared to empty vector, *p* = 0.6). Out of the two Fox/Sox (FS) binding sites, FS1 (the more 5’) appears to be critical as activity was significantly reduced with its disruption (Figure 5). Though enhancer activity appeared modestly reduced with ΔFS2, the reduction was not significant (Figure 5D). In contrast, disruption of the two Sox-only binding sites (ΔS) increased enhancer activity, suggesting they play an inhibitory role in regulating *GARR* activity. Yet, in the absence of the Fox/Sox binding sites, no increase in activity was detected when Sox-only binding sites were also disrupted (ΔAll). Similar results were observed in the chicken elbow bioassay (Figure 5C). Taken together, these results indicate that the Fox/Sox binding sites are necessary for *GARR* activity, while the Sox-only sites are involved with repression of activity.

### Lmx1b binding sites in *GARR* are critical for dorsal enhancer activity

**FIGURE 6:**
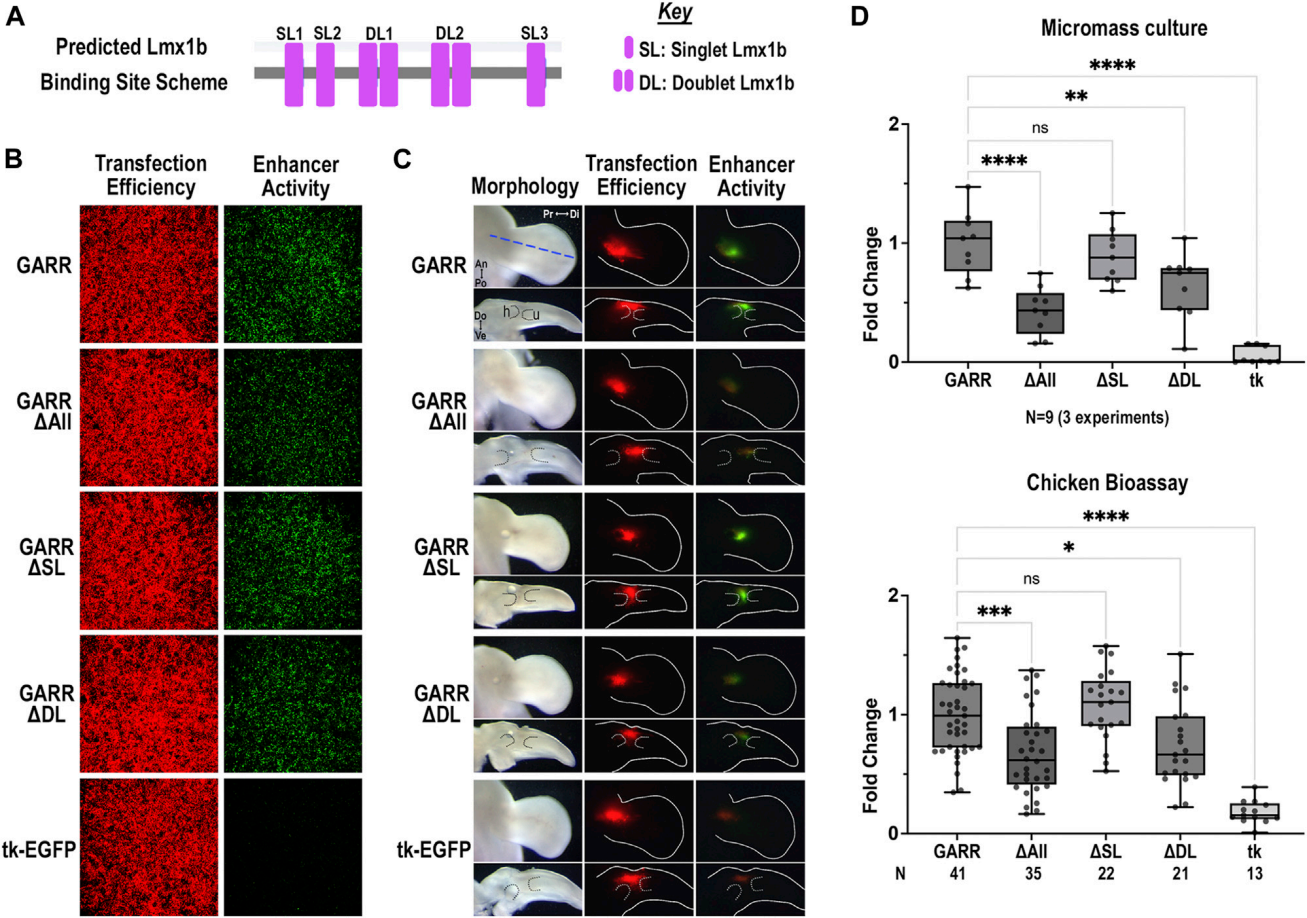
Predicted Lmx1b binding sites enhance dorsal *GARR* activity **(A)** Binding site scheme for Lmx1b transcription factor (not to scale). **(B)** Micromass cultures transfected with *GARR*-GFP reporter shows that mutation of all predicted Lmx1b binding sites (ΔAll) or the doublet Lmx1b binding sites (ΔDL) significantly reduces enhancer activity. **(C)** Targeted transfection of mutant *GARR*-GFP reporter constructs in the elbow with dorsal (top panel) and cross-section (bottom panel) views confirms results in micromass. Blue dashed line shows plane of section for the longitudinal cross-sections. Dotted lines demarcate cartilage. **(D)** Semi-quantification of fluorescence images from micromass cultures and chick enhancer bioassay in B and C respectively. Box plots show range, interquartile range, and median with statistical significance determined by one-way ANOVA and Tukey’s HSD (α = 0.05). N indicates the number of experiments or embryos. Abbreviations used: An (anterior), Di (distal), Do (dorsal), h (humerus), Po (posterior), and Pr (proximal), tk (tk-EGFP), u (ulna), and Ve (ventral).

The mouse *GARR* sequence has two doublet Lmx1b binding sites and 3 single Lmx1b sites. Mutating the two highly conserved doublet Lmx1b binding sites (ΔDL) was sufficient to reduce activity; however, the greatest loss of enhancer activity was achieved with mutation of all Lmx1b binding sites (ΔAll) (Figure 6). In both the micromass cultures (Figure 6B) and the chicken elbow joint (Figure 6C), enhancer activity was markedly reduced in the ΔAll or ΔDL constructs. Consistent with findings from micromass culture, disruptions of the three single Lmx1b sites (ΔSL) did not significantly affect enhancer activity in the dorsal limb. The single Lmx1b sites (SL) collectively contribute to overall enhancer activity. There is a significant difference between the ΔDL construct when compared to the empty vector (mean difference of 0.5849 and *p*-value < 0.0001). However, in the absence of the SL sites (ΔSL), enhancer activity was similar to wildtype (Figure 6D). These findings suggest a critical role for Lmx1b binding sites in the dorsal regulation of *Gdf5* through *GARR* in the elbow joint.

### Spatial regulation of *GARR* activity along the dorsoventral axis

**FIGURE 7:**
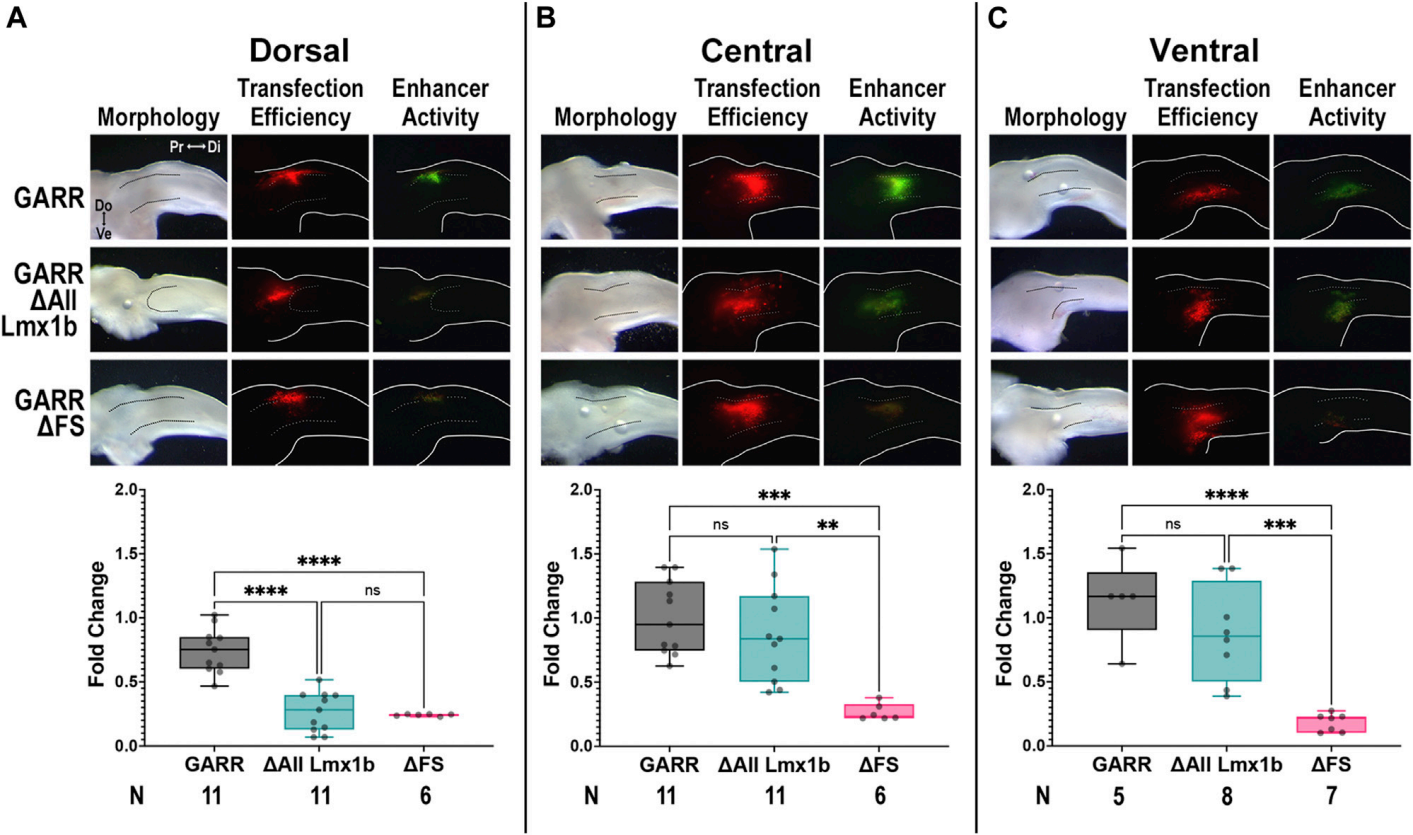
Predicted Lmx1b binding sites are not necessary for ventral enhancer activityTargeted transfection of wildtype *GARR* (top panel), and constructs with disruption of all predicted Lmx1b binding sites (ΔAll Lmx1b, middle panel) and the two Fox/Sox binding sites (ΔFS, bottom panel), in the **(A)** dorsal, **(B)** central, and **(C)** ventral limb around the elbow shows ΔAll Lmx1b retains central and ventral limb activity whereas ΔFS loses activity along the entire dorsoventral axis. Dotted lines show region of condensing cartilage. Box plots show semi-quantification of fluorescent images and one-way ANOVA statistical analysis with Tukey’s HSD (α = 0.05). N indicates the number of embryos Abbreviations used: Di (distal), Do (dorsal), and Pr (proximal), and Ve (ventral).

Since Lmx1b is only expressed in the dorsal mesoderm (Figure 3A), we evaluated the activity of *GARR* enhancer constructs all the Lmx1b binding sites disrupted (ΔAll Lmx1b) along the dorsoventral axis of the chicken limb (Figure 7). Activity was assessed in the dorsal, central (cartilage condensation shown by dotted lines), and ventral limb mesoderm at the level of the elbow. Since neither *Fox* nor *Sox* expression have a dorsoventral bias, we also evaluated the ΔFS construct which has a near complete loss of activity in the elbow. In dorsal transfections, *GARR* activity was robust, while ΔAll Lmx1b and ΔFS had almost no activity (Figure 7A). In the central and ventral limb, activity persisted in both the wildtype and the ΔAll Lmx1b constructs (Figures 7B, C). In contrast, no substantial activity was detected with ΔFS construct. Consistent with the expression pattern of *Lmx1b*, disruption of its binding sites alters the dorsal enhancer activity only, suggesting Lmx1b is crucial for normal *GARR*-mediated *Gdf5* expression in the dorsal elbow joint. Loss of activity along the entire dorsoventral axis from disruption of the shared Fox/Sox sites suggests a more fundamental role for these transcription factors in regulating *GARR* activity that is independent of dorsoventral position. These data corroborate findings from Lmx1b knockout mice, where the level of *Gdf5* expression during elbow development is reduced to less than 50% of wild type expression (Feenstra et al., 2012).

## Discussion

The articulating ends of limb bones have precise morphology and asymmetry that ensures proper joint function. Slight alterations in the shape of the articulating joint surfaces are more likely to compromise joint function and with time joint integrity (Muthuirulan et al., 2021). Gdf5 is well-recognized as an important modulator of cartilage and joint formation. Over and under expression of Gdf5 leads to corresponding changes in bone length, and causes joint dysmorphology or fusion (Francis-West et al., 1999; Merino et al., 1999; Storm and Kingsley, 1999; Buxton et al., 2001; Settle et al., 2003; Masuya et al., 2007; Degenkolbe et al., 2013). Recent work demonstrated a correlation between decreasing *Gdf5* levels and abnormal joint morphology with subsequent instability (Muthuirulan et al., 2021). Consequently, a defined and consistent expression of Gdf5 is required during early joint development to ensure proper joint morphology. Several CRMs, including *GARR*, that contribute to the spatiotemporal regulation and maintenance of *Gdf5* expression in the limb have been identified (Chen et al., 2016; Haro et al., 2017). Interestingly, disrupted *GARR* (R4) function affects the knee more than the hip suggesting this CRM is a primary regulator for stylopod-zeugopod articulations (Muthuirulan et al., 2021). Nevertheless, the transcription factors that mediate the regulation of *Gdf5* through these regulatory modules are largely unknown.

One major finding in this study is that Fox/Sox binding sites (FS1 and FS2) within *GARR* are required for enhancer activity. A loss of these sites caused a near-absence of enhancer activity in micromass cultures and chick bioassays, demonstrating their essential role in *GARR*-mediated *Gdf5* expression. As master regulators of cartilage development, both Fox and Sox transcription factors may also regulate *Gdf5* to modify the structure of articulating surfaces at joints. Gdf5 has been shown to be expressed in the condensing cartilage, perichondrium and interzone where it promotes chondrogenesis through promoting cell attachment and its action on cells in the developing epiphyseal plate (Buxton et al., 2001). Cells expressing *Sox9* give rise to *Gdf5*-expressing cells (Shwartz et al., 2016). However, there is considerable overlap in the expression patterns of the *Sox* and *Fox* transcription factors implying other Sox and Fox transcription factors could play a role in contributing to *Gdf5*-expressing cells. Those we evaluated in this study have a demonstrated role in chondrogenesis (Lefebvre et al., 2001; Kan et al., 2013; Kato et al., 2015; Liu and Lefebvre, 2015; Yoshida et al., 2015; Zhao et al., 2015; Xu P. et al., 2018; Almubarak et al., 2021; Xu et al., 2021). As such many of these transcription factors are expressed in condensing cartilage, interzone and perichondrium overlapping Gdf5. Additionally, except for FoxP1/2/4 and FoXC2, other Sox and Fox transcription factors investigated were upregulated in all Gdf5 expressing cells compared to Gdf5 non-expressing chondrogenic cells (Supplementary Table S3). Even in non-chondrogenic cells (Col2A1-), all Fox and Sox transcription factors were increased in Gdf5 expressing cells suggesting a fundamental role in Gdf5 regulation. The Fox/Sox sites that are required for *GARR* activity are predicted to bind to Sox4/6, FoxC1/2, and FoxP1/2/4 transcription factors and not Sox9. This is interesting because a ChIP-seq to Sox9 identified this region in rib cartilage (Ohba et al., 2015). Thus, a combination of these transcription factors might be necessary to initiate and maintain *Gdf5*-expressing cells. Sox9 may also act indirectly to regulate *Gdf5* or through other *Gdf5*-associated CRMs.

Surprisingly, in the absence of the two Sox-only binding sites (S1 and S2) within *GARR*, enhancer activity was increased suggesting a role in enhancer repression. Thus, Sox transcription factors could repress *Gdf5* expression through a reduction in *GARR* activity. This signifies a potential dual role for Sox transcription factors in promoting as well as restricting *Gdf5* expression through *GARR*. Differential expression of *Sox* transcription factors in OA provides further support for a dual role of Sox transcription factors. Different Sox transcription factors may regulate *GARR* and *Gdf5* in disparate ways: downregulation of Sox5/6/9 is associated with OA progression (Lee and Im, 2011), while Sox4 and 11 are upregulated in OA (Xu et al., 2019; Ahmed and Alzahrani, 2023). Thus, downregulation of Gdf5 in OA pathology implies Sox5/6/9 as positive regulators, whereas Sox4/11 are negative regulators of Gdf5. Sox4 is one of the transcription factors predicted to bind S1 and could repress *GARR* activity and thus *Gdf5* expression. Alternatively, the same Sox transcription factors could play a role in both activating and inhibiting *GARR* activity with their function contingent on environment. Sox4 and Sox6 are both predicted to bind activating FS sites as well as repressive Sox-only sites. It is possible these two factors inhibit as well as promote *GARR*-associated *Gdf5* expression.

Alternatively, Fox and Sox transcription factors could have different roles in *GARR*-mediated *Gdf5* expression. A loss of *FoxP2* in mouse embryos leads to abnormal knee joint development that result in progressive OA later in life (Xu S. et al., 2018). The features are similar to the abnormal knees that develop due to the loss or mutation of *GARR* (R4) and reduced *Gdf5* expression (Muthuirulan et al., 2021). These mice also develop OA later in life. Fox transcription factors (such as FoxP2) could be enhancing *GARR* activity through the FS sites, while Sox transcription factors inhibit *GARR* activity through Sox-only sites. Together, the action of these transcription factors could initiate, confine, and maintain *Gdf5* expression in joints. This is particularly relevant since it is anticipated that a combination of factors is required to initiate and localize *Gdf5* expression. For example, although not all *Sox9* expressing cells co-express *Gdf5*, the compressed *Gdf5*-positive interzone cells that identify presumptive joints originate from the Sox/Fox-expressing cartilage anlagen. Additionally, our findings suggest that upregulation of *Gdf5* via the *GARR* enhancer requires the Fox/Sox binding sites consistent with the cells’ anlagen origin. Therefore, although these transcription factors may not be sufficient alone for *Gdf5* expression, our data suggest they are required for *Gdf5* expression. Varying combinations of Fox and Sox transcription factors in different regions of cartilage may lead to the formation of different transcriptional complexes and consequently differentially regulate the level or localization of *Gdf5* expression.

Another key finding from our study is the requirement for Lmx1b binding sites within *GARR* to facilitate dorsal enhancer activity. Lmx1b is required for limb dorsalization and upregulates *Gdf5* during elbow/knee development (Feenstra et al., 2012). Moreover, using an Lmx1b-targeted ChIP-seq we demonstrated that Lmx1b binds to *GARR* (previously identified as LBI443) and several other potential regulatory regions around *Gdf5* (Haro et al., 2017). Lmx1b directs the formation of joints that are asymmetrical along the extensor-flexor (dorsoventral) axis. In the absence of Lmx1b, the ends of articulating bones (particularly of elbows and knees) become more symmetrical and fail to support normal movement and stance confirming the importance of dorsoventral asymmetry for normal joint movement and function (Haro et al., 2021). Our findings that Lmx1b binding sites significantly contribute to *GARR* activity provides a mechanistic contribution to the spatial regulation of *Gdf5* by Lmx1b along the dorsoventral axis. The absence of dorsal *GARR* activity in the ΔAll Lmx1b construct suggests that Lmx1b recruits Gdf5 to help modify dorsal joint structures, particularly at the stylopod-zeugopod articulation. The presence of ventral *GARR* activity in ΔAll Lmx1b constructs indicates that other transcription factors, perhaps Sox/Fox or even Hox and Barx-like transcription factors (with similar binding sites to Lmx1b), are required to position, establish, or maintain Gdf5 to form synovial joints. In humans, haploinsufficiency of *LMX1B* causes nail-patella syndrome (NPS) and incomplete limb dorsalization. NPS often presents with elbow and knee abnormalities including hypoplastic or absent patellae. In addition, patients with NPS often develop degenerative arthritis further linking the regulation of Gdf5 to OA (Lucas et al., 1966; Lachiewicz and Herndon, 1997; Chen et al., 1998; Sweeney et al., 2003; Curbo et al., 2019).

Contrary to our expectation, the highly conserved Osr2 site was not required for *GARR* activity. Previous findings show that Osr transcription factors are essential to normal synovial joint formation; in the absence of these transcription factors, several joint fusions occurred (Gao et al., 2011). How Osr is related to joint formation has not been extensively studied. Robust *Osr2* expression in the joints and a positive correlation of *Gdf5* with *Osr2* across multiple scRNA-seq experiments shown in our analyses, support a role for Osr2 in synovial joint development and possibly *Gdf5* regulation (Figure 2 and Supplementary Figure S4). However, disruption of the binding site showed no significant change in enhancer activity. This assay system may not be adequate to determine the spatial changes in Osr2-mediated *GARR* activity. It is also possible that Osr2 regulation of *Gdf5* may be achieved through other regulatory elements or in joints other than the elbow. Alternatively, the influence of Osr2 on joint formation may be indirect through the regulation of other factors.

In summary, we have characterized some key transcription factor binding sites within *GARR*, a CRM of *Gdf5*, that differentially regulate its activity: two Fox/Sox sites that are required for *GARR* activity and two Sox sites that appear to suppress activity. We have also validated the micromass culture method as a tool for studying the mechanism behind *GARR* regulation of *Gdf5*. These findings point toward complex differential regulation of *Gdf5* by the Fox and Sox transcription factor families that are linked to cartilage anlagen and joint formation. We have also identified binding sites through which Lmx1b can modulate *Gdf5* to support the formation of dorsoventrally asymmetrical joints. It is also important to note that disruption or loss in the *GARR* (R4) sequence primarily affects the stylopod-zeugopod articulation, pointing to an important role for this regulatory element in elbow/knee development. Thus, the characterization of this CRM also provides a novel tool to further investigate the differential regulation of *Gdf5* that correlates with variations in elbow/knee morphology during both development and degeneration.

## Data Availability

The published datasets that were analyzed for scRNA-seq were obtained from the EN-CODE consortium under ENCSR713GIS or from the GEO database under accession numbers GSE151985, GSE142425, and GSE145657. Other datasets and materials used for this study are available upon request.
