# Supplementary material for: Sox, Fox, and Lmx1b binding sites differentially regulate a Gdf5-Associated regulatory region during elbow development
Source: Front Cell Dev Biol. 2023 Jul 10;11:1215406. doi: 10.3389/fcell.2023.1215406 (PMC10364121; doi:10.3389/fcell.2023.1215406)
Supplement: Supplementary file 1 [file Table1.docx]

Supplementary Material

Lmx1b, Sox and Fox Transcription Factors Regulate Gdf5 expression through a downstream enhancer element during Joint Development.

Spatial and Disparate Regulation of Gdf5-Associated Regulatory Region through Conserved Fox, Sox, and Lmx1B Binding Sites in the developing Elbow Joint

Ruth-Love Yeboah ^1^, Jessie Lin ^1^, Matthew Shankel ^1,2^ ^*^, Charmaine U Pira ^1^, Allen M Cooper^1^, Nicole Sandoval ^1^, Van-Dai Ly ^1^, Kerby C Oberg ^1*^

*** Correspondence:** Kerby C Oberg: [koberg@llu.edu](mailto:koberg@llu.edu)

# Supplementary Tables and Figures

## Supplementary Tables

| **Chicken Probe Primers** | |
| --- | --- |
| PRIMER NAME | PRIMER SEQUENCE |
| cCOL2A1 F | GCCACCCTCAAATCCCTCAA |
| cCOL2A1 R | ATCACCGTCTTGCCCCAT |
| cFOXC1 F | TGGCCTCCTGGTACCTCAAC |
| cFOXC1 R | TCCGTGTTAACGACCCTTCC |
| cFOXC2 F | CGGAAGATTTCGGCTGTT |
| cFOXC2 R | GTCCGAGACGGAATAGCGAG |
| cFOXP1 F | GACAACAGCCAACCACAGTCCA |
| cFOXP1 R | GGGCGGGGGAACAAAAGCAA |
| cFOXP2 F | AGCCATTCGAAGACGACATT |
| cFOXP2 R | ACATGGATAGAATGTATGTGAGG |
| cFOXP4 F | AGCACGAGTACCAAAAGCGA |
| cFOXP4 R | GGAAGGAGGGACAACTTCGG |
| cGDF5 F | CCATGGATTTAGCACCG |
| cGDF5 R | CCTGTCAAAAGTTTCCAGTTC |
| cLMX1B F | GGATCGCTTTCTGATGAGG |
| cLMX1B R | CGCTGAGAATTTTGTTGCTCC |
| cOSR1 F | ATCCTTCCCTCCAGCTTACC |
| cOSR1 R | GAGATTCAGCACTTAAGTTTGGC |
| cOSR2pr F | ACCACTGGACGTTGGGCT |
| cOSR2 R | ATGTGCAGCGTTTTGTGG |
| cSOX4 F | CGCCCTCCGGATGCGAAAGAG |
| cSOX4PR R | AGCGCCATCTGTCCGCTTCG |
| cSOX5 F | CTCTCCTCATATGCCAACTCT |
| cSOX5 R | GGCTGCTTCTCTAGGTTTGT |
| CSOX6 F | GTTCATTCCGTCAACAATGG |
| CSOX6 R | TGTAGTTTAGCTGCAGAGCCAT |
| cSox9 F | CTCCGTCTCTGCCGGCTTTACT |
| cSox9 R | CTGCTGATGCCGTAGGTA |
| cSox11 F | CGGTCAAGTGCGTTTTCG |
| cSox11 R | GCGAAGAAGTCTTTTGGCAG |

**Supplementary Table 1.** Table of primers used for chicken probe generation.

| ***Gdf5*+ vs *Gdf5*-** | | | | | | | | |  |
| --- | --- | --- | --- | --- | --- | --- | --- | --- | --- |
| **Analysis of Dataset from He P, Williams et al. nature 2022** | | | | | | | | |  |
| **Feature ID** | **P-value** | **Q-value** | **FDR**  **step up** | **Bonferroni** | **Ratio** | **Fold change** | **LSMean**  **(*Gdf5*+)** | **LSMean**  **(*Gdf5*-)** | |
| ***Col2a1*** | 0.00E+00 | 0.00E+00 | 0.00E+00 | 0.00E+00 | 3.41 | 3.41 | 234.14 | 68.76 | |
| ***Foxc1*** | 0.00E+00 | 0.00E+00 | 0.00E+00 | 0.00E+00 | 5.36 | 5.36 | 86.34 | 16.10 | |
| ***Foxc2*** | 8.46E-115 | 7.44E-113 | 1.30E-112 | 2.97E-110 | 2.67 | 2.67 | 15.53 | 5.82 | |
| ***Foxp1*** | 1.58E-78 | 8.17E-77 | 1.43E-76 | 5.55E-74 | 1.22 | 1.22 | 166.61 | 136.22 | |
| ***Foxp2*** | 6.64E-321 | 2.29E-318 | 4.02E-318 | 2.33E-316 | 1.87 | 1.87 | 80.60 | 43.14 | |
| ***Foxp4*** | 3.13E-175 | 4.51E-173 | 7.90E-173 | 1.10E-170 | 1.71 | 1.71 | 62.73 | 36.64 | |
| ***Gdf5*** | 0.00E+00 | 0.00E+00 | 0.00E+00 | 0.00E+00 | 337.02 | 337.02 | 396.53 | 1.18 | |
| ***Lmx1b*** | 3.40E-18 | 3.43E-17 | 6.01E-17 | 1.19E-13 | 1.41 | 1.41 | 27.30 | 19.41 | |
| ***Osr1*** | 1.42E-89 | 8.63E-88 | 1.51E-87 | 4.98E-85 | 1.32 | 1.32 | 113.05 | 85.33 | |
| ***Osr2*** | 1.69E-90 | 1.04E-88 | 1.82E-88 | 5.94E-86 | 1.73 | 1.73 | 129.51 | 74.66 | |
| ***Sox11*** | 2.84E-304 | 9.33E-302 | 1.63E-301 | 9.97E-300 | 1.34 | 1.34 | 1076.78 | 801.74 | |
| ***Sox4*** | 0.00E+00 | 0.00E+00 | 0.00E+00 | 0.00E+00 | 1.80 | 1.80 | 903.35 | 502.59 | |
| ***Sox5*** | 0.00E+00 | 0.00E+00 | 0.00E+00 | 0.00E+00 | 4.66 | 4.66 | 76.01 | 16.31 | |
| ***Sox6*** | 0.00E+00 | 0.00E+00 | 0.00E+00 | 0.00E+00 | 4.81 | 4.81 | 70.27 | 14.60 | |
| ***Sox9*** | 0.00E+00 | 0.00E+00 | 0.00E+00 | 0.00E+00 | 3.61 | 3.61 | 374.59 | 103.75 | |

**Supplementary Table 2.** ANOVA differential expression analysis comparing *Gdf5* expressing (*Gdf5*+) and Gdf5 non-expressing (*Gdf5*-) cells. Fold change and adjusted p-values shown in Figure 3 are reported here. Data obtained from He P, Williams et al. nature 2022^1^.

| ***Gdf5*+ vs *Col2A1*+, *Gdf5*-**  **Analysis of Dataset from He P, Williams et al. nature 2022** | | | | | | | | | |
| --- | --- | --- | --- | --- | --- | --- | --- | --- | --- |
| **Feature ID** | **P-value** | **Q-value** | **FDR**  **step up** | **Bonferroni** | **Ratio** | **Fold change** | **LSMean**  **(*Gdf5*+)** | **LSMean**  **(*Col2A1*+, *Gdf5*-)** |  |
| ***Col2a1*** | 0.00E+00 | 0.00E+00 | 0.00E+00 | 0.00E+00 | 0.58 | -1.72 | 248.57 | 426.78 |  |
| ***Foxc1*** | 0.00E+00 | 0.00E+00 | 0.00E+00 | 0.00E+00 | 1.92 | 1.92 | 88.31 | 46.03 |  |
| ***Foxc2*** | 4.00E-07 | 3.74E-06 | 5.36E-06 | 1.40E-02 | 1.04 | 1.04 | 15.83 | 15.19 |  |
| ***Foxp1*** | 2.13E-03 | 9.33E-03 | 1.34E-02 | 1.00E+00 | 1.04 | 1.04 | 166.31 | 160.17 |  |
| ***Foxp2*** | 1.53E-02 | 4.93E-02 | 7.06E-02 | 1.00E+00 | 0.97 | -1.03 | 81.36 | 83.93 |  |
| ***Foxp4*** | 3.52E-01 | 4.51E-01 | 6.46E-01 | 1.00E+00 | 1.12 | 1.12 | 63.45 | 56.51 |  |
| ***Gdf5*** | 0.00E+00 | 0.00E+00 | 0.00E+00 | 0.00E+00 | 402.37 | 402.37 | 399.89 | 0.99 |  |
| ***Lmx1b*** | 2.03E-02 | 6.19E-02 | 8.86E-02 | 1.00E+00 | 1.25 | 1.25 | 27.27 | 21.90 |  |
| ***Osr1*** | 5.49E-90 | 1.19E-87 | 1.71E-87 | 1.93E-85 | 1.57 | 1.57 | 112.83 | 71.99 |  |
| ***Osr2*** | 2.08E-39 | 1.43E-37 | 2.05E-37 | 7.29E-35 | 1.65 | 1.65 | 132.62 | 80.47 |  |
| ***Sox11*** | 1.71E-71 | 2.67E-69 | 3.82E-69 | 6.00E-67 | 1.59 | 1.59 | 1069.59 | 670.83 |  |
| ***Sox4*** | 1.88E-91 | 4.20E-89 | 6.01E-89 | 6.61E-87 | 1.61 | 1.61 | 909.30 | 564.69 |  |
| ***Sox5*** | 2.42E-139 | 9.56E-137 | 1.37E-136 | 8.49E-135 | 1.57 | 1.57 | 78.47 | 50.08 |  |
| ***Sox6*** | 1.63E-192 | 1.03E-189 | 1.47E-189 | 5.73E-188 | 1.59 | 1.59 | 72.36 | 45.58 |  |
| ***Sox9*** | 9.15E-104 | 2.39E-101 | 3.42E-101 | 3.22E-99 | 1.35 | 1.35 | 385.18 | 285.76 |  |

**Supplementary Table 3.** ANOVA differential expression analysis comparing *Gdf5* expressing (*Gdf5*+) and *Col2*+, *Gdf5*- cells. Fold change and adjusted p-values of transcription factors of interest are reported. Data obtained from He P, Williams et al. nature 2022^1^.

| ***Gdf5*+ vs *Col2A1*-, *Gdf5*-**  **Analysis of Dataset from He P, Williams et al. nature 2022 (Limbs)** | | | | | | | | |
| --- | --- | --- | --- | --- | --- | --- | --- | --- |
| **Feature ID** | **P-value** | **Q-value** | **FDR**  **step up** | **Bonferroni** | **Ratio** | **Fold change** | **LSMean**  **(*Gdf5*+)** | **LSMean**  **(*Col2A1*-, *Gdf5*-)** |
| ***Col2a1*** | 0.00E+00 | 0.00E+00 | 0.00E+00 | 0.00E+00 | 182.26 | 182.26 | 248.57 | 1.36 |
| ***Foxc1*** | 0.00E+00 | 0.00E+00 | 0.00E+00 | 0.00E+00 | 8.44 | 8.44 | 88.31 | 10.46 |
| ***Foxc2*** | 2.91E-190 | 3.76E-188 | 6.69E-188 | 1.02E-185 | 3.90 | 3.90 | 15.83 | 4.06 |
| ***Foxp1*** | 5.09E-105 | 3.02E-103 | 5.37E-103 | 1.79E-100 | 1.26 | 1.26 | 166.31 | 131.71 |
| ***Foxp2*** | 0.00E+00 | 0.00E+00 | 0.00E+00 | 0.00E+00 | 2.29 | 2.29 | 81.36 | 35.46 |
| ***Foxp4*** | 1.97E-258 | 3.78E-256 | 6.72E-256 | 6.92E-254 | 1.93 | 1.93 | 63.45 | 32.90 |
| ***Gdf5*** | 0.00E+00 | 0.00E+00 | 0.00E+00 | 0.00E+00 | 330.22 | 330.22 | 399.89 | 1.21 |
| ***Lmx1b*** | 3.64E-22 | 3.77E-21 | 6.69E-21 | 1.28E-17 | 1.44 | 1.44 | 27.27 | 18.94 |
| ***Osr1*** | 2.08E-77 | 8.78E-76 | 1.56E-75 | 7.31E-73 | 1.28 | 1.28 | 112.83 | 87.84 |
| ***Osr2*** | 1.42E-113 | 9.15E-112 | 1.62E-111 | 4.97E-109 | 1.80 | 1.80 | 132.62 | 73.57 |
| ***Sox11*** | 0.00E+00 | 0.00E+00 | 0.00E+00 | 0.00E+00 | 1.29 | 1.29 | 1069.59 | 826.38 |
| ***Sox4*** | 0.00E+00 | 0.00E+00 | 0.00E+00 | 0.00E+00 | 1.85 | 1.85 | 909.30 | 490.90 |
| ***Sox5*** | 0.00E+00 | 0.00E+00 | 0.00E+00 | 0.00E+00 | 7.89 | 7.89 | 78.47 | 9.95 |
| ***Sox6*** | 0.00E+00 | 0.00E+00 | 0.00E+00 | 0.00E+00 | 8.25 | 8.25 | 72.36 | 8.77 |
| ***Sox9*** | 0.00E+00 | 0.00E+00 | 0.00E+00 | 0.00E+00 | 5.54 | 5.54 | 385.18 | 69.49 |

**Supplementary Table 4.** ANOVA differential expression analysis comparing *Gdf5* expressing (*Gdf5*+) and *Col2*-, *Gdf5*- cells. Fold change and adjusted p-values of transcription factors of interest are reported. Data obtained from He P, Williams et al. nature 2022^1^.

| ***Col2A1*+, *Gdf5*+ vs *Col2A1*-, *Gdf5*+**  **Analysis of Dataset from He P, Williams et al. nature 2022 (Limbs)** | | | | | | | | |
| --- | --- | --- | --- | --- | --- | --- | --- | --- |
| **Feature ID** | **P-value** | **Q-value** | **FDR**  **step up** | **Bonferroni** | **Ratio** | **Fold change** | **LSMean**  **(*Col2A1*+, *Gdf5*+)** | **LSMean**  **(*Col2A1*-, *Gdf5*+)** |
| ***Col2a1*** | 0.00E+00 | 0.00E+00 | 0.00E+00 | 0.00E+00 | 552.67 | 552.67 | 496.25 | 0.90 |
| ***Foxc1*** | 0.00E+00 | 0.00E+00 | 0.00E+00 | 0.00E+00 | 2.23 | 2.23 | 121.99 | 54.63 |
| ***Foxc2*** | 1.09E-88 | 2.45E-86 | 3.18E-86 | 3.81E-84 | 1.99 | 1.99 | 21.09 | 10.57 |
| ***Foxp1*** | 5.56E-10 | 1.01E-08 | 1.31E-08 | 1.95E-05 | 0.94 | -1.06 | 161.33 | 171.30 |
| ***Foxp2*** | 6.15E-127 | 2.16E-124 | 2.80E-124 | 2.16E-122 | 1.39 | 1.39 | 94.54 | 68.19 |
| ***Foxp4*** | 4.69E-76 | 8.64E-74 | 1.12E-73 | 1.65E-71 | 1.48 | 1.48 | 75.63 | 51.26 |
| ***Gdf5*** | 2.12E-71 | 3.59E-69 | 4.66E-69 | 7.46E-67 | 1.34 | 1.34 | 457.50 | 342.28 |
| ***Lmx1b*** | 9.97E-01 | 7.71E-01 | 1.00E+00 | 1.00E+00 | 0.96 | -1.04 | 26.75 | 27.80 |
| ***Osr1*** | 1.23E-05 | 1.21E-04 | 1.57E-04 | 4.32E-01 | 0.94 | -1.07 | 109.18 | 116.48 |
| ***Osr2*** | 2.13E-192 | 1.16E-189 | 1.50E-189 | 7.50E-188 | 2.35 | 2.35 | 186.02 | 79.21 |
| ***Sox11*** | 1.22E-14 | 3.47E-13 | 4.51E-13 | 4.28E-10 | 0.79 | -1.26 | 946.04 | 1193.13 |
| ***Sox4*** | 8.89E-67 | 1.37E-64 | 1.77E-64 | 3.12E-62 | 1.25 | 1.25 | 1011.35 | 807.25 |
| ***Sox5*** | 0.00E+00 | 0.00E+00 | 0.00E+00 | 0.00E+00 | 3.34 | 3.34 | 120.80 | 36.15 |
| ***Sox6*** | 0.00E+00 | 0.00E+00 | 0.00E+00 | 0.00E+00 | 2.96 | 2.96 | 108.18 | 36.53 |
| ***Sox9*** | 0.00E+00 | 0.00E+00 | 0.00E+00 | 0.00E+00 | 2.79 | 2.79 | 566.90 | 203.46 |

**Supplementary Table 5.** ANOVA differential expression analysis comparing *Gdf5* expressing cells that are chondrocytes (Col2A+, *Gdf5+)* and those that are non-chondrocytes (*Col2A1-, Gdf5+*). Fold change and adjusted p-values of transcription factors of interest are reported. Data obtained from He P, Williams et al. nature 2022^1^.

| ***Col2A1*+, *Gdf5*+ vs *Col2A1*+*Gdf5*-**  **Analysis of Dataset from He P, Williams et al. nature 2022 (Limbs)** | | | | | | | | |
| --- | --- | --- | --- | --- | --- | --- | --- | --- |
| **Feature ID** | **P-value** | **Q-value** | **FDR**  **step up** | **Bonferroni** | **Ratio** | **Fold change** | **LSMean**  **( *Col2A1*+, *Gdf5*+)** | **LSMean**  **( *Col2A1*+, *Gdf5*-)** |
| ***Col2a1*** | 6.54E-112 | 2.09E-109 | 2.80E-109 | 2.30E-107 | 1.16 | 1.16 | 496.25 | 426.78 |
| ***Foxc1*** | 0.00E+00 | 0.00E+00 | 0.00E+00 | 0.00E+00 | 2.65 | 2.65 | 121.99 | 46.03 |
| ***Foxc2*** | 3.64E-15 | 8.96E-14 | 1.20E-13 | 1.28E-10 | 1.39 | 1.39 | 21.09 | 15.19 |
| ***Foxp1*** | 1.30E-09 | 1.93E-08 | 2.60E-08 | 4.57E-05 | 1.01 | 1.01 | 161.33 | 160.17 |
| ***Foxp2*** | 8.89E-35 | 6.15E-33 | 8.26E-33 | 3.12E-30 | 1.13 | 1.13 | 94.54 | 83.93 |
| ***Foxp4*** | 2.27E-31 | 1.36E-29 | 1.83E-29 | 7.97E-27 | 1.34 | 1.34 | 75.63 | 56.51 |
| ***Gdf5*** | 0.00E+00 | 0.00E+00 | 0.00E+00 | 0.00E+00 | 460.33 | 460.33 | 457.50 | 0.99 |
| ***Lmx1b*** | 7.05E-02 | 1.85E-01 | 2.48E-01 | 1.00E+00 | 1.22 | 1.22 | 26.75 | 21.90 |
| ***Osr1*** | 2.59E-39 | 2.07E-37 | 2.78E-37 | 9.09E-35 | 1.52 | 1.52 | 109.18 | 71.99 |
| ***Osr2*** | 1.72E-169 | 8.80E-167 | 1.18E-166 | 6.03E-165 | 2.31 | 2.31 | 186.02 | 80.47 |
| ***Sox11*** | 5.72E-21 | 2.14E-19 | 2.88E-19 | 2.01E-16 | 1.41 | 1.41 | 946.04 | 670.83 |
| ***Sox4*** | 2.43E-149 | 1.06E-146 | 1.42E-146 | 8.54E-145 | 1.79 | 1.79 | 1011.35 | 564.69 |
| ***Sox5*** | 0.00E+00 | 0.00E+00 | 0.00E+00 | 0.00E+00 | 2.41 | 2.41 | 120.80 | 50.08 |
| ***Sox6*** | 0.00E+00 | 0.00E+00 | 0.00E+00 | 0.00E+00 | 2.37 | 2.37 | 108.18 | 45.58 |
| ***Sox9*** | 0.00E+00 | 0.00E+00 | 0.00E+00 | 0.00E+00 | 1.98 | 1.98 | 566.90 | 285.76 |

**Supplementary Table 6.** ANOVA differential expression analysis comparing *Gdf5* expressing (*Gdf5*+) and Gdf5 non-expressing (*Gdf5*-) chondrocytes (*Col2A1*+). Fold change and adjusted p-values of transcription factors of interest are reported. Data obtained from He P, Williams et al. nature 2022^1^.

| **Col2A1-, Gdf5+ vs Col2A1-, Gdf5-**  **Analysis of Dataset from He P, Williams et al. nature 2022 (Limbs)** | | | | | | | | |
| --- | --- | --- | --- | --- | --- | --- | --- | --- |
| **Feature ID** | **P-value** | **Q-value** | **FDR**  **step up** | **Bonferroni** | **Ratio** | **Fold change** | **LSMean**  **(*Gdf5*+)** | **LSMean**  **(*Gdf5-*)** |
| ***Col2a1*** | 0.00E+00 | 0.00E+00 | 0.00E+00 | 0.00E+00 | 0.66 | -1.52 | 0.90 | 1.36 |
| ***Foxc1*** | 2.45E-316 | 3.27E-313 | 4.78E-313 | 8.61E-312 | 5.22 | 5.22 | 54.63 | 10.46 |
| ***Foxc2*** | 7.23E-16 | 1.57E-14 | 2.30E-14 | 2.54E-11 | 2.60 | 2.60 | 10.57 | 4.06 |
| ***Foxp1*** | 4.33E-33 | 2.23E-31 | 3.26E-31 | 1.52E-28 | 1.30 | 1.30 | 171.30 | 131.71 |
| ***Foxp2*** | 3.37E-81 | 5.37E-79 | 7.84E-79 | 1.18E-76 | 1.92 | 1.92 | 68.19 | 35.46 |
| ***Foxp4*** | 9.66E-38 | 6.04E-36 | 8.84E-36 | 3.39E-33 | 1.56 | 1.56 | 51.26 | 32.90 |
| ***Gdf5*** | 0.00E+00 | 0.00E+00 | 0.00E+00 | 0.00E+00 | 282.64 | 282.64 | 342.28 | 1.21 |
| ***Lmx1b*** | 3.64E-13 | 6.36E-12 | 9.29E-12 | 1.28E-08 | 1.47 | 1.47 | 27.80 | 18.94 |
| ***Osr1*** | 3.45E-65 | 4.11E-63 | 6.01E-63 | 1.21E-60 | 1.33 | 1.33 | 116.48 | 87.84 |
| ***Osr2*** | 1.31E-04 | 6.62E-04 | 9.68E-04 | 1.00E+00 | 1.08 | 1.08 | 79.21 | 73.57 |
| ***Sox11*** | 2.54E-266 | 2.77E-263 | 4.05E-263 | 8.91E-262 | 1.44 | 1.44 | 1193.13 | 826.38 |
| ***Sox4*** | 5.76E-208 | 4.32E-205 | 6.32E-205 | 2.02E-203 | 1.64 | 1.64 | 807.25 | 490.90 |
| ***Sox5*** | 5.55E-102 | 1.17E-99 | 1.71E-99 | 1.95E-97 | 3.63 | 3.63 | 36.15 | 9.95 |
| ***Sox6*** | 6.74E-187 | 3.95E-184 | 5.77E-184 | 2.37E-182 | 4.16 | 4.16 | 36.53 | 8.77 |
| ***Sox9*** | 0.00E+00 | 0.00E+00 | 0.00E+00 | 0.00E+00 | 2.93 | 2.93 | 203.46 | 69.49 |

**Supplementary Table 7.** ANOVA differential expression analysis comparing *Gdf5* expressing (*Gdf5*+) and Gdf5 non-expressing (*Gdf5*-) non-chondrocytes (*Col2A1*-). Fold change and adjusted p-values of transcription factors of interest are reported. Data obtained from He P, Williams et al. nature 2022^1^.

**Analysis of Dataset from He P, Williams et al. nature 2022 (Limbs)**

|  | ***Gdf5* Cells only** | ***EF* Cells only** | **Both *Gdf5* and *EF* Cells** | **Other Cells** | **All Cells** |  |
| --- | --- | --- | --- | --- | --- | --- |
| *Gdf5* | 13573 |  |  | 115480 | 129053 |  |
| *Col2A1* | 7182 | 18295 | 6391 | 97185 | 129053 |  |
| *FoxC1* | 8744 | 8384 | 4829 | 107096 | 129053 |  |
| *FoxC2* | 12547 | 2990 | 1026 | 112490 | 129053 |  |
| *FoxP1* | 5783 | 48868 | 7790 | 66612 | 129053 |  |
| *FoxP2* | 8381 | 21122 | 5192 | 94358 | 129053 |  |
| *FoxP4* | 8816 | 25050 | 4757 | 90430 | 129053 |  |
| *Lmx1b* | 11526 | 14762 | 2047 | 100718 | 129053 |  |
| *Osr1* | 8037 | 33630 | 5536 | 81850 | 129053 |  |
| *Osr2* | 9092 | 28662 | 4481 | 86818 | 129053 |  |
| *Sox5* | 9302 | 10179 | 4271 | 105301 | 129053 |  |
| *Sox6* | 9293 | 8867 | 4280 | 106613 | 129053 |  |
| *Sox9* | 4822 | 31212 | 8751 | 84268 | 129053 |  |
| *Sox4* | 907 | 91768 | 12666 | 23712 | 129053 |  |
| *Sox11* | 1234 | 89005 | 12339 | 26475 | 129053 |  |
|  | | | | | | |
|  | ***Gdf5* Cells only** | ***EF* Cells only** | **Both *Gdf5* and *EF* Cells** | **Other Cells** |  |  |
| *Gdf5* | 10.5 | 0.0 | 0.0 | 89.5 |  |  |
| *Col2A1* | 5.6 | 14.2 | 5.0 | 75.3 |  |  |
| *FoxC1* | 6.8 | 6.5 | 3.7 | 83.0 |  |  |
| *FoxC2* | 9.7 | 2.3 | 0.8 | 87.2 |  |  |
| *FoxP1* | 4.5 | 37.9 | 6.0 | 51.6 |  |  |
| *FoxP2* | 6.5 | 16.4 | 4.0 | 73.1 |  |  |
| *FoxP4* | 6.8 | 19.4 | 3.7 | 70.1 |  |  |
| *Lmx1b* | 8.9 | 11.4 | 1.6 | 78.0 |  |  |
| *Osr1* | 6.2 | 26.1 | 4.3 | 63.4 |  |  |
| *Osr2* | 7.0 | 22.2 | 3.5 | 67.3 |  |  |
| *Sox5* | 7.2 | 7.9 | 3.3 | 81.6 |  |  |
| *Sox6* | 7.2 | 6.9 | 3.3 | 82.6 |  |  |
| *Sox9* | 3.7 | 24.2 | 6.8 | 65.3 |  |  |
| *Sox4* | 0.7 | 71.1 | 9.8 | 18.4 |  |  |
| *Sox11* | 1.0 | 69.0 | 9.6 | 20.5 |  |  |

**Supplementary Table 8.** Table of cells coexpressing factors of interest as well as *Gdf5* in tSNE plots reported in Figure 3 and Supplementary Figure 6. The lower table represents the numbers as percentages. Data obtained from He P, Williams et al. nature 2022^1^.

**Analysis of Dataset from Bian Q, et al. (Knees)**

|  | ***Gdf5* Cells only** | ***EF* Cells only** | **Both *Gdf5* and *EF* Cells** | **Other Cells** | **All Cells** |  |
| --- | --- | --- | --- | --- | --- | --- |
| *Gdf5* | 1321 |  |  | 12214 | 13535 |  |
| *Col2A1* | 426 | 3624 | 895 | 8590 | 13535 |  |
| Lmx1b | 1240 | 417 | 81 | 11797 | 13535 |  |
| *Osr1* | 957 | 3538 | 364 | 8676 | 13535 |  |
| *Osr2* | 808 | 2090 | 513 | 10124 | 13535 |  |
| *FoxC1* | 907 | 904 | 414 | 11310 | 13535 |  |
| *FoxC2* | 1254 | 244 | 67 | 11970 | 13535 |  |
| *FoxP1* | 563 | 5097 | 758 | 7117 | 13535 |  |
| *FoxP2* | 721 | 2451 | 600 | 9763 | 13535 |  |
| *FoxP4* | 919 | 1906 | 402 | 10308 | 13535 |  |
| *Sox5* | 795 | 1156 | 526 | 11058 | 13535 |  |
| *Sox6* | 821 | 1081 | 500 | 11133 | 13535 |  |
| *Sox9* | 317 | 3657 | 1004 | 8557 | 13535 |  |
| *Sox4* | 181 | 8473 | 1140 | 3741 | 13535 |  |
| *Sox11* | 312 | 6781 | 1009 | 5433 | 13535 |  |
|  | | | | | | |
|  | ***Gdf5* Cells only** | ***EF* Cells only** | **Both *Gdf5* and *EF* Cells** | **Other Cells** |  |  |
| *Gdf5* | 9.8 | 0.0 | 0.0 | 90.2 |  |  |
| *Col2A1* | 3.1 | 6.6 | 26.8 | 63.5 |  |  |
| *FoxC1* | 9.2 | 0.6 | 3.1 | 87.2 |  |  |
| *FoxC2* | 7.1 | 2.7 | 26.1 | 64.1 |  |  |
| *FoxP1* | 6.0 | 3.8 | 15.4 | 74.8 |  |  |
| *FoxP2* | 6.7 | 3.1 | 6.7 | 83.6 |  |  |
| *FoxP4* | 9.3 | 0.5 | 1.8 | 88.4 |  |  |
| *Lmx1b* | 4.2 | 5.6 | 37.7 | 52.6 |  |  |
| *Osr1* | 5.3 | 4.4 | 18.1 | 72.1 |  |  |
| *Osr2* | 6.8 | 3.0 | 14.1 | 76.2 |  |  |
| *Sox5* | 5.9 | 3.9 | 8.5 | 81.7 |  |  |
| *Sox6* | 6.1 | 3.7 | 8.0 | 82.3 |  |  |
| *Sox9* | 2.3 | 7.4 | 27.0 | 63.2 |  |  |
| *Sox4* | 1.3 | 8.4 | 62.6 | 27.6 |  |  |
| *Sox11* | 2.3 | 7.5 | 50.1 | 40.1 |  |  |

**Supplementary Table 9.** Table of cells coexpressing factors of interest as well as *Gdf5* in tSNE plots reported in Supplementary Figure 7. The lower table represents the numbers as percentages. Data obtained from Bian Q, et al. Development 2020**^2^**

| **Dataset** | **No. of cells** | **Gdf5+ Cells** | **Col2A1+ cells** | **Lmx1b cells** | **Model** | **Tissue type** | **Stages** |
| --- | --- | --- | --- | --- | --- | --- | --- |
| He P. 2020^1^ | 129053 | 13573 | 24686 | 16809 | Mouse | Limbs | E11-15 |
| Bian Q. 2020^2^ | 13535 | 1321 | 4519 | 498 | Mouse | Knees | E12.5-15.5 |
| Kelly NH. 2020^3^ | 7715 | 774 | 5987 | 357 | Mouse | Hindlimbs | E11-15 |
| Desanlis I 2020^4^ | 21972 | 2585 | 12237 | 8818 | Mouse | Forelimbs | E10.5-12.5 |
| Feregrino C. 2022^5^ | 17591 | 527 | 2304 | 861 | Chicken | Hindlimb Autopod | HH25-31 |
| Esteves de Lima, J. 2021^6^ | 37481 | 65 | 488 | 1369 | Chicken | Forelimbs | HH23-36 |

**Supplementary Table 10.** Summary table of datasets used for single cell analysis in Figure 3, Supplementary Figures 6 and 7 as well as for the supplementary excel file (Supplementary Table 12).

| **GARR mutagenesis Primers** | |
| --- | --- |
| PRIMER NAME | PRIMER  SEQUENCE |
| S1 NheI | GACCCGTCCCTGGCCCGCTCGTGTGCTAGCTCTTTGCCC |
| S2 mut | CCGCTCGTGTTTGTTTTCTTTGCCGTCTGTGCCTGCC |
| S3 SacI | CTTGTGCCTGCCTCTGCTATAACAGAGCTCGTATGTGTTAAGTAATCC |
| S4 mut | GTGCATGGCAAATATATTATAACTCATGGACTCGTCTGAAAAATGAACGGAT |
| OSRMut2 | GCAGTCATCATTTAACCCTCTGATTTCGTCTGTTTAGTTTAT |
| OSRSacI | CAGGAATTGCAGTCATCATTTAACCCTCTGATTTGAGCTCTTTAGTTTAT |
| L3D1 mut | CCTTAAGGTAACATTTTCAGTATGTCGTCTGAAGTGCATGGCAAATATATTATAACTCA |
| L4D2 XhoI | TTATGTACTCAATAAAAATTCTCGAGAGTCGTCTCAGGAATTGCAGTCATCATTTAACCC |
| LS1 XhoI | TTAAGAATACTTCCATCTCGAGAATGTGTGCCTTGCAAGATCCC |
| LS2 SacI | CTGTCGGCTGTCTCATTCAGAGCTCTGCTGATTAGTAACCC |
| LS3 XoI | CCCTCTGATTTCTTCTGTTTAGTCTCGAGTTAAACATTCAGTAATAGCA |

**Supplementary Table 11.** Table of primers used for site directed mutagenesis. Letters in red are changed from the original sequence, while those in green are neighboring binding site sequence that was kept the same as the original sequence.

## Supplementary Figures
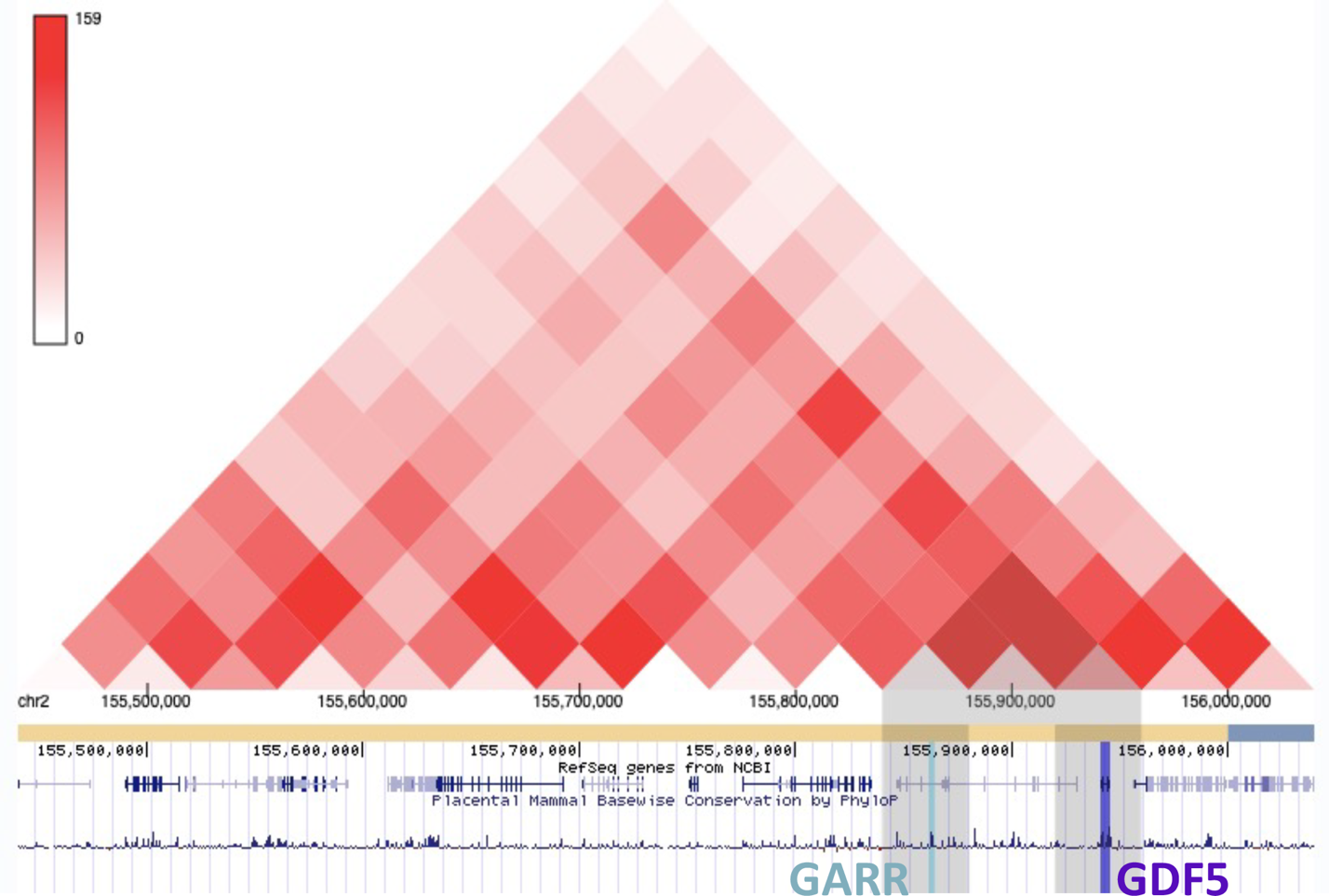


**Supplementary Figure 1.** *Gdf5* Topologically Associating Domain generated from mouse embryonic stem cell^7^ shows *GARR* and *Gdf5* have a higher frequency of interaction (regions highlighted in grey). Image generated using 3D genome browser^8^.


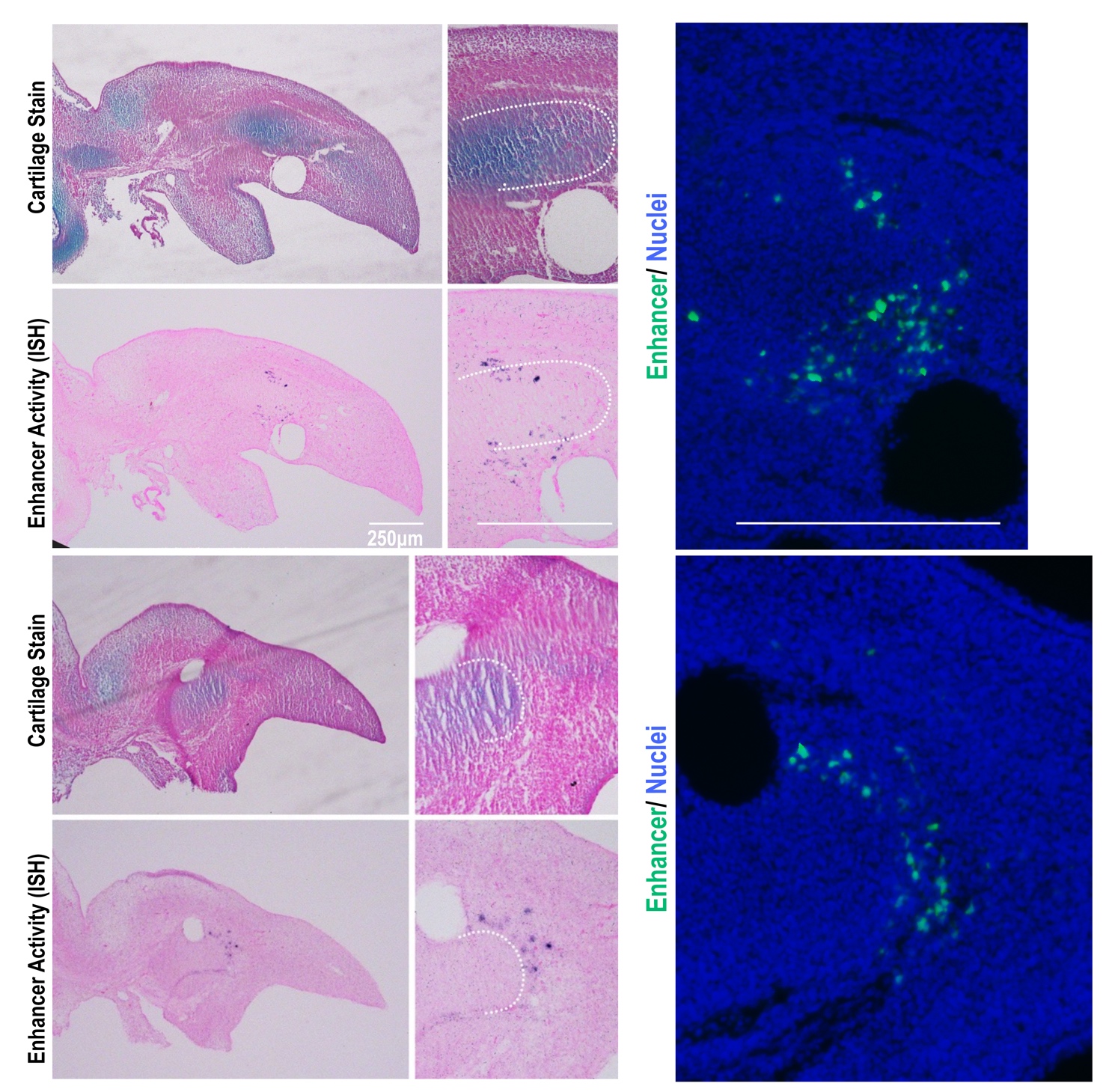
**Supplementary Figure 2.** *GARR* Enhancer Activity in perichondral and joint space. Cartilage stain was done using alcian blue and nuclear fast red counterstain, enhancer activity is shown by *GFP* ISH and IHC (Hoechst dye used as nuclear dye).


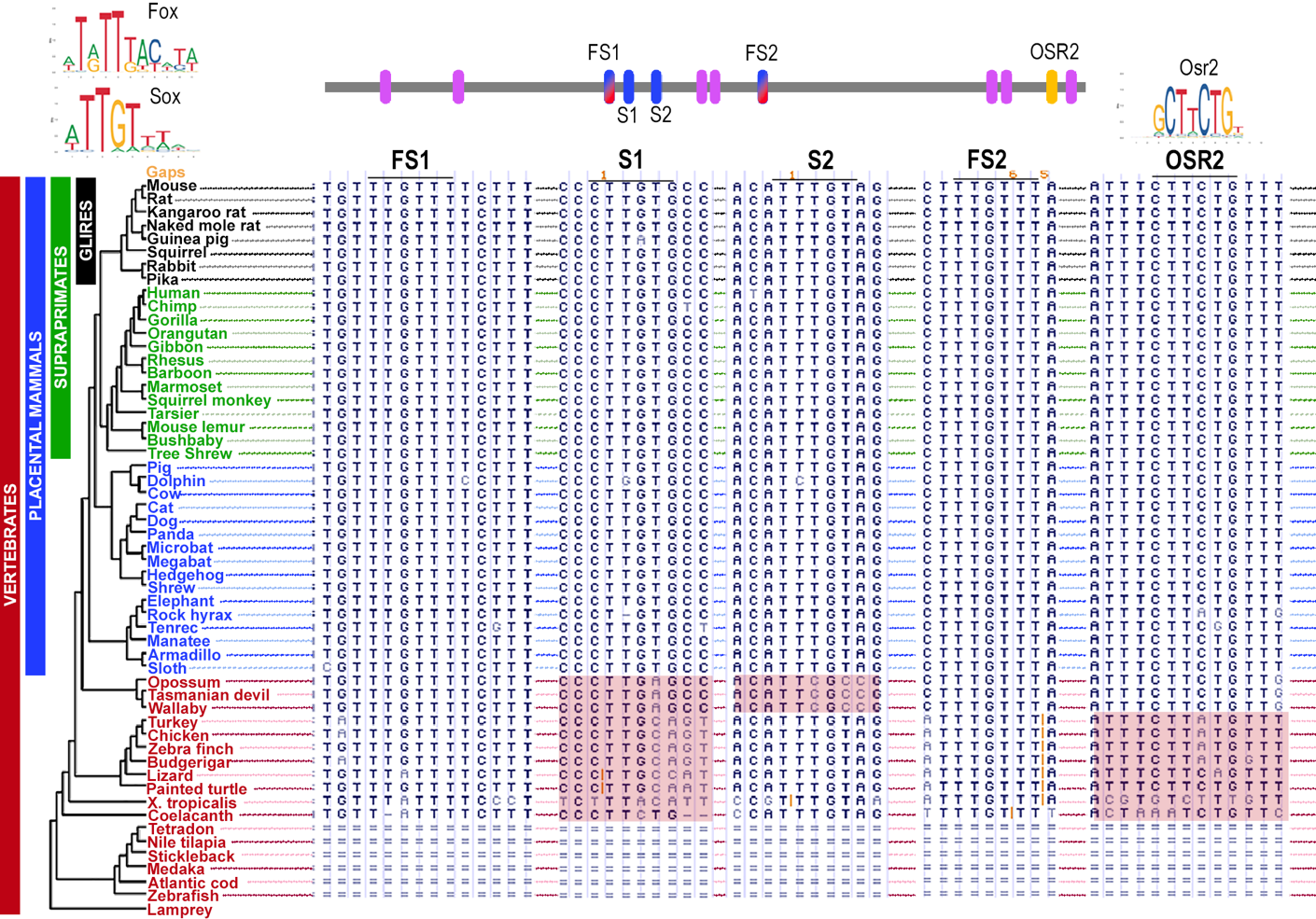


**Supplementary Figure 3.** Fox and Sox binding sites conservation in common vertebrates shows *GARR* is not conserved in bony fish. Core sequence of binding sites is not conserved for two Sox only binding sites is not conserved in birds and marsupials while the core sequence for Ors2 is not conserved in birds only. (Phylogenetic tree modeled after USCS genome browser)


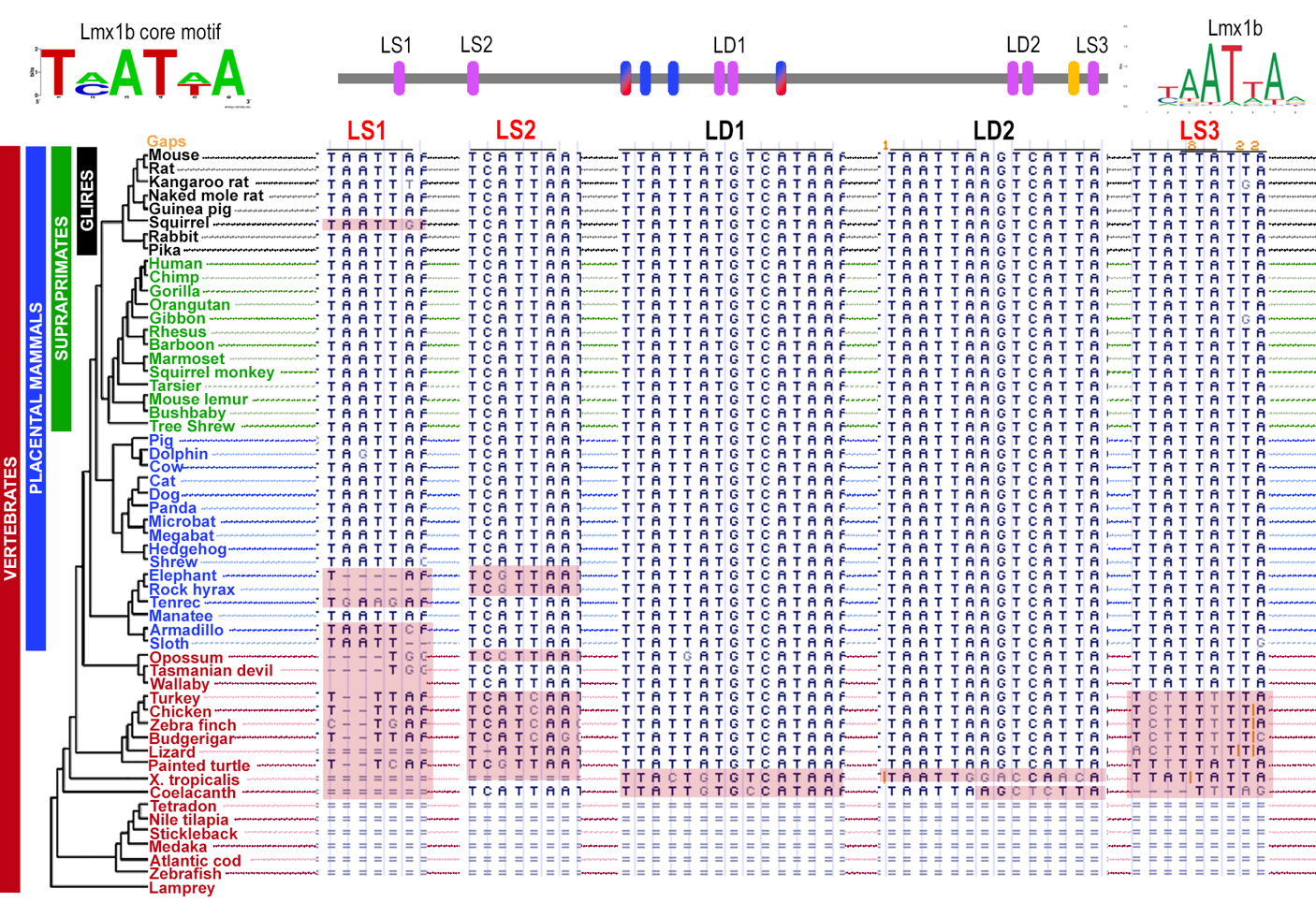


**Supplementary Figure 4** Lmx1b binding sites conservation in common vertebrates shows *GARR* is not conserved in bony fish. Core sequence of Lmx1b binding sites in the frog and coelacanth. Single Lmx1b sites are not conserved in birds and in some mammals including the squirrel, elephant and marsupials. (Phylogenetic tree modeled after USCS genome browser)


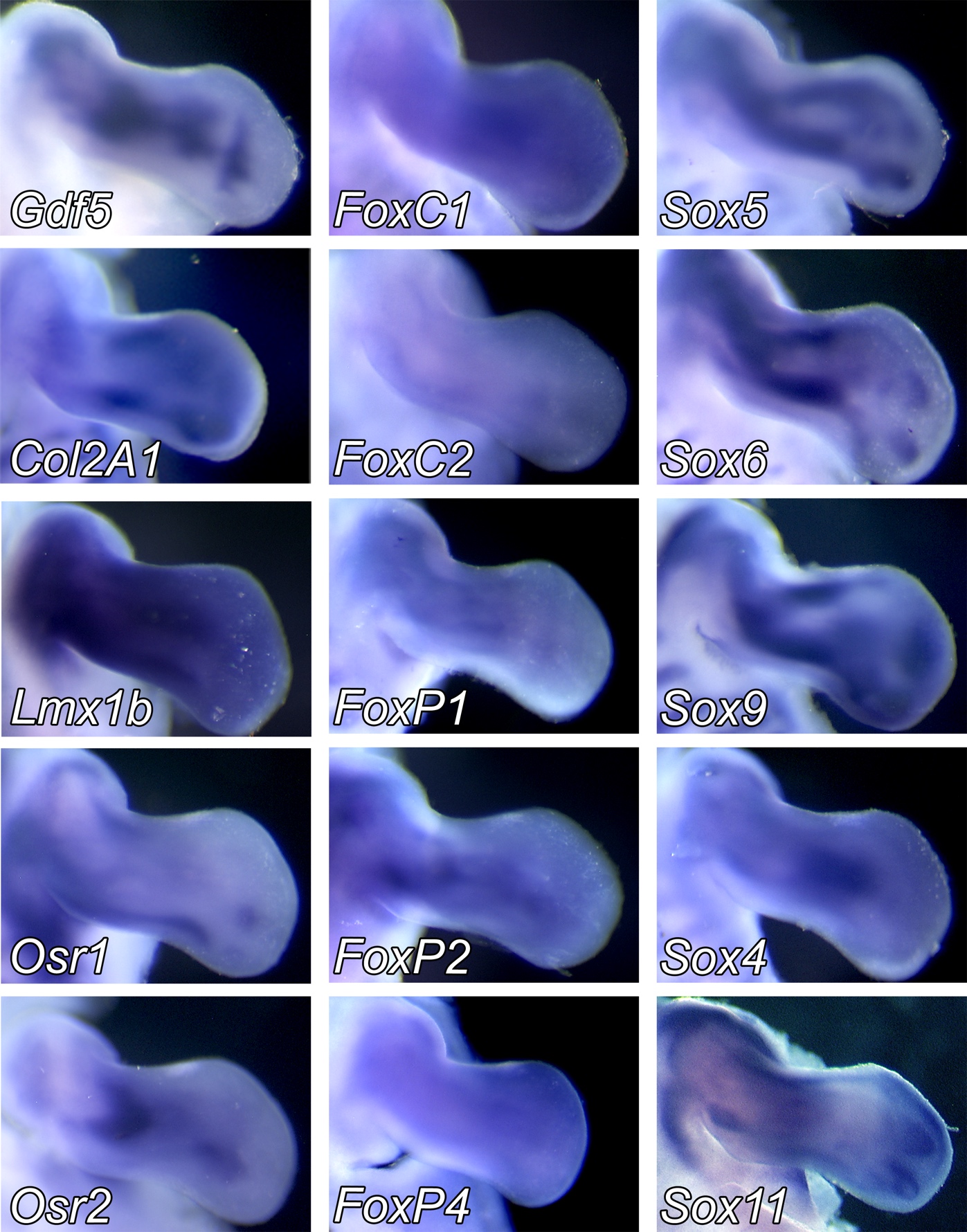


**Supplementary Figure 5.** Whole mount ISH of HH27 chicken embryos showing expression of factor of interest as well as *Gdf5*.


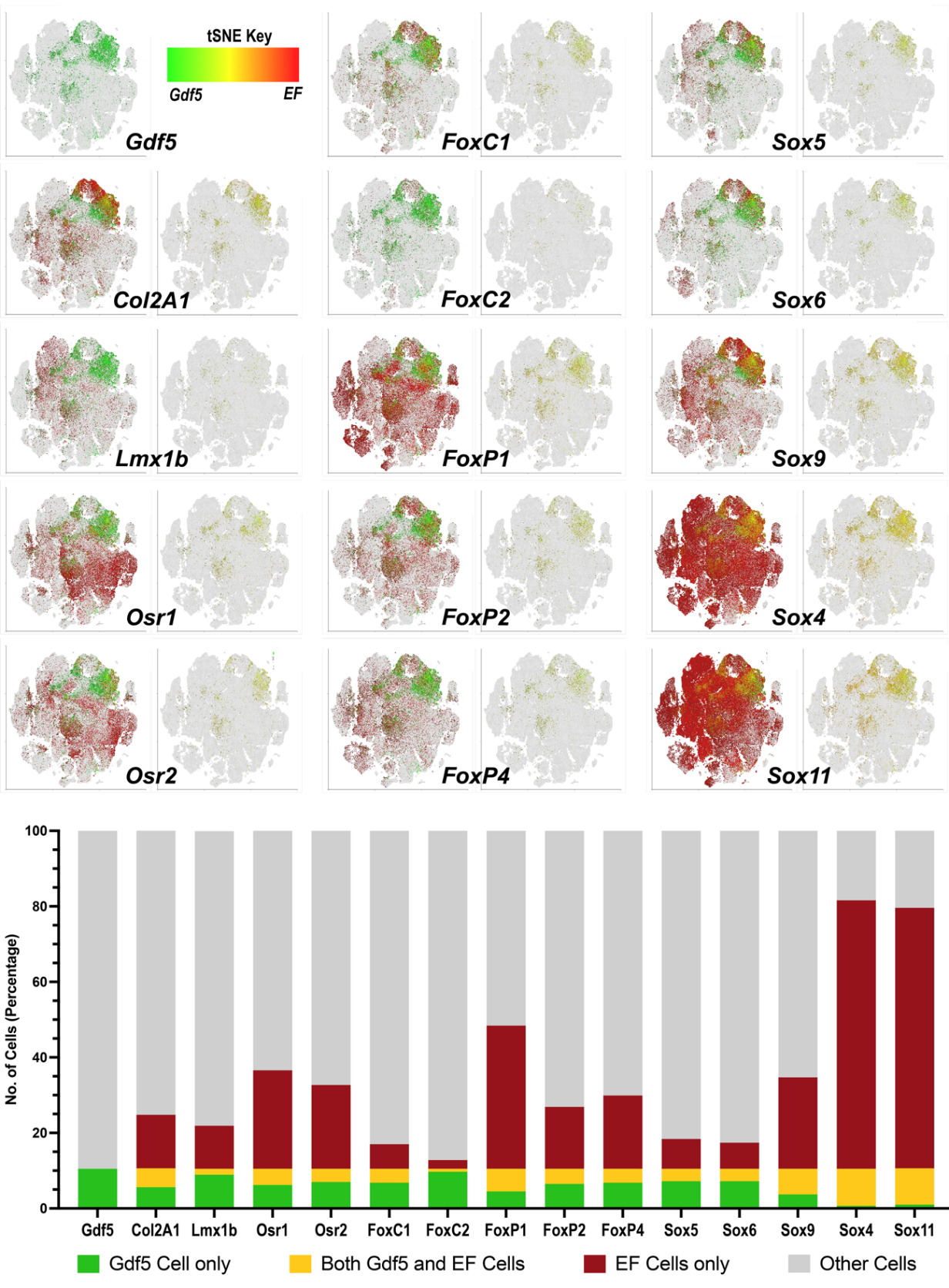


**Supplementary Figure 6.** Full tSNE plots showing distribution of cells coexpressing *Gdf5* and factors of interest shown in Figure 2. The second plot in each pair for a specific factor, highlights the cells that are coexpressing *Gdf5* and the factor of interest only (yellow cells). Stacked bar graphs illustrate the percentage of each group in the plots shown. Data from He, P., Williams, B.A., Trout, D. et al. 2020^1^.

**
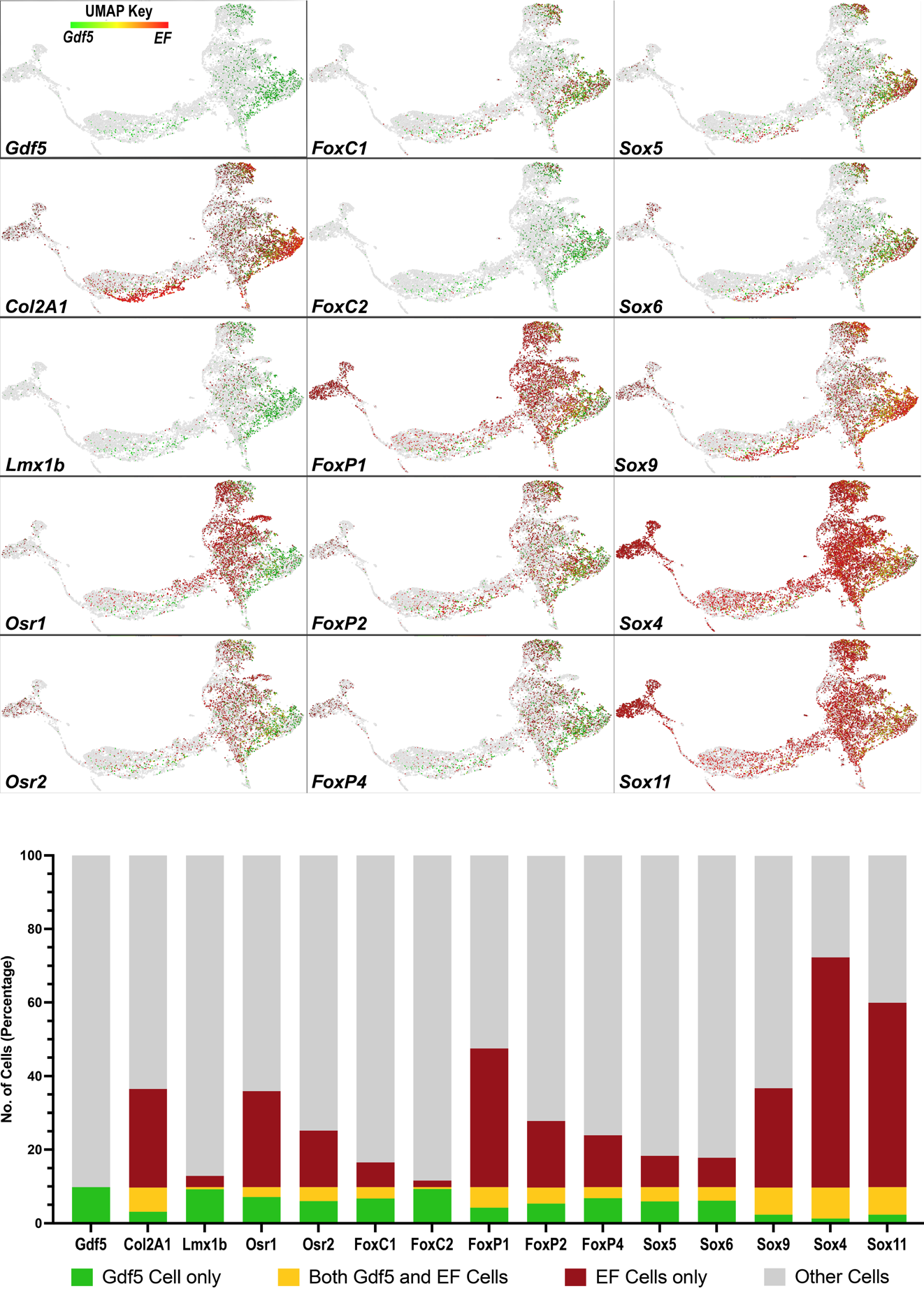
**

**Supplementary Figure 7.** Full UMAP plots showing distribution of cells coexpressing *Gdf5* and factors of interest in mouse knee scRNA-seq data. Stacked bar graphs illustrate the percentage of each group in the plots shown. Data from Bian Q, et al. Development 2020^2^.

References:

1. He, P. *et al.* The changing mouse embryo transcriptome at whole tissue and single-cell resolution. *Nature* **583**, 760-767 (2020).

2. Bian, Q. *et al.* A single cell transcriptional atlas of early synovial joint development. *Development* **147** (2020).

3. Kelly, N.H., Huynh, N.P.T. & Guilak, F. Single cell RNA-sequencing reveals cellular heterogeneity and trajectories of lineage specification during murine embryonic limb development. *Matrix Biol* **89**, 1-10 (2020).

4. Desanlis, I., Paul, R. & Kmita, M. Transcriptional Trajectories in Mouse Limb Buds Reveal the Transition from Anterior-Posterior to Proximal-Distal Patterning at Early Limb Bud Stage. *J Dev Biol* **8** (2020).

5. Feregrino, C. & Tschopp, P. Assessing evolutionary and developmental transcriptome dynamics in homologous cell types. *Dev Dyn* **251**, 1472-1489 (2022).

6. Esteves de Lima, J. *et al.* Unexpected contribution of fibroblasts to muscle lineage as a mechanism for limb muscle patterning. *Nat Commun* **12**, 3851 (2021).

7. Dixon, J.R. *et al.* Topological domains in mammalian genomes identified by analysis of chromatin interactions. *Nature* **485**, 376-380 (2012).

8. Wang, Y. *et al.* The 3D Genome Browser: a web-based browser for visualizing 3D genome organization and long-range chromatin interactions. *Genome Biol* **19**, 151 (2018).
